# Long noncoding RNA MALAT-1: A versatile regulator in cancer progression, metastasis, immunity, and therapeutic resistance

**DOI:** 10.1016/j.ncrna.2024.01.015

**Published:** 2024-02-01

**Authors:** Dexin Xu, Wenhai Wang, Duo Wang, Jian Ding, Yunan Zhou, Wenbin Zhang

**Affiliations:** a Department of Orthopedics, Jilin Province FAW General Hospital, Changchun, 130000, China; b Department of Cardiology, Jilin Province FAW General Hospital, Changchun, 130000, China; c Department of Geriatrics, Jilin Province FAW General Hospital, Changchun, 130000, China; d Department of Electrodiagnosis, Jilin Province FAW General Hospital, Changchun, 130000, China

**Keywords:** lncRNA MALAT-1, Cancer immunity, Chemoresistance, Immunotherapy, Tumor microenvironment

## Abstract

Long noncoding RNAs (lncRNAs) are RNA transcripts longer than 200 nucleotides that do not code for proteins but have been linked to cancer development and metastasis. Metastasis-associated lung adenocarcinoma transcript 1 (MALAT-1) influences crucial cancer hallmarks through intricate molecular mechanisms, including proliferation, invasion, angiogenesis, apoptosis, and the epithelial-mesenchymal transition (EMT). The current article highlights the involvement of MALAT-1 in drug resistance, making it a potential target to overcome chemotherapy refractoriness. It discusses the impact of MALAT-1 on immunomodulatory molecules, such as major histocompatibility complex (MHC) proteins and PD-L1, leading to immune evasion and hindering anti-tumor immune responses. MALAT-1 also plays a significant role in cancer immunology by regulating diverse immune cell populations. In summary, MALAT-1 is a versatile cancer regulator, influencing tumorigenesis, chemoresistance, and immunotherapy responses. Understanding its precise molecular mechanisms is crucial for developing targeted therapies, and therapeutic strategies targeting MALAT-1 show promise for improving cancer treatment outcomes. However, further research is needed to fully uncover the role of MALAT-1 in cancer biology and translate these findings into clinical applications.

## 1 Introduction

Cancer poses a substantial global health threat and stands as the second-leading cause of death in the United States [1]. Globally, there were 9.96 million fatalities due to cancer and 19.29 million newly identified individuals with cancer in 2020 [2]. Considering impressive developments in medical technology designed to fight cancer, the burden associated with it is still quite significant [3,4]. As a result, it is crucial to delve into factors related to cancer risk to determine at-risk groups and provide them with protection. Growing research indicates that genetic factors significantly influence the progression of cancer, as well as well-established environmental risk factors [5,6] (see Table 1).

**Table 1:** The mechanistic role of MALAT-1 in various types of cancer.

Cancer type	Study setting	MALAT-1 expression	Molecular target	Result	Ref
HCC	HCC tissues (metastatic and non-metastatic) and HepG2 cell	Upregulated	miRNA-613 ↓	Promote HCC metastasis	[65]
HCC	HCC tissues and cell lines	Upregulated	miRNA-146a ↓	Inhibits the apoptosis and autophagy of HCC cell	[40]
HCC	HCC and corresponding nontumor liver tissues and human HCC cell lines	Upregulated	miR-140-5p ↓ Aurora-A Signaling ↑	Contributes to sorafenib resistance	[222]
HCC	HCC cell line and mice model	Upregulated	miR-22-3p ↓	Promote HCC progression	[223]
HCC	HCC cells and tissues	Upregulated	BRG1 ↑	Promote inflammation-related HCC.	[54]
HCC	HCC cells and tissues	Upregulated	miR-140 ↓	Promotes HCC immunosuppression and angiogenesis	[49]
HCC	HCC patient samples and cells	Upregulated	miR-125a-3p ↓	Promote HCC progression	[56]
HCC	HCC cell lines	Upregulated	miRNA-200a ↓	Regulates HCC development under hypoxia	[55]
HCC	Animal experiments and cell culture	Upregulated	SRSF1 ↑ mTOR ↑	Promotes HCC development	[224]
CRC		Upregulated	β-catenin signaling pathway ↑	Promotes CRC metastasis	[93]
CRC	TCGA database, tumor tissues, and CRC cell lines	Upregulated		Promotes radioresistance and aggressive malignancy in CRC	[70]
CRC	CRC tissues and normal mucosal samples	Upregulated	miR-378a-3p ↓	Promotes CRC progression	[225]
CRC	CRC tissues and paired adjacent noncancerous tissues, and human CRC cell lines	Upregulated	EZH2 ↑ miR-218 ↓	Implicated in the poor response to oxaliplatin-based chemotherapy and promotes chemoresistance in CRC	[72]
CRC	CRC specimens and cell culture	Upregulated	miR-26a-5p ↓	Promotes CRC progression	[71]
CRC	Tissue specimens and human CRC cell lines	Upregulated	miR-508-5p ↓	Promotes CRC progression	[78]
CRC	HCC patient samples, CRC cell lines, and mice model	Upregulated	miR-106b-5p ↓	Promote the invasion and metastasis of CRC	[226]
Colon cancer	Tissue specimens	Upregulated	Wnt/β-catenin signaling pathway ↑	Inhibits apoptosis and promote cell proliferation	[38]
Gastric cancer	Gastric cancer cell line SGC7901	Upregulated	miR-383-5p ↓ DDIT4 ↑	Promote the migration, invasion, and proliferation, and inhibit apoptosis	[227]
Gastric cancer	Plasma and tissue sample	Upregulated	PI3K/AKT pathway ↑	Promotes invasion and migration proliferation of gastric cancer	[228]
Gastric cancer	Human gastric cancer cell lines and BALB/C nude mice	Upregulated	Cancer-associated fibroblasts ↑	Promotes gastric cancer progression	[229]
Gastric cancer	Gastric cancer tissues and normal tumor-adjacent tissues and cell culture	Upregulated	miR-22-3p ↓	Modulates oxaliplatin resistance in gastric cancer	[230]
Gastric cancer	Gastric cancer cell lines	Upregulated	SF2/ASF ↑	Promotes cell proliferation in gastric cancer	[102]
Prostate cancer	Prostate cancer cell lines	Upregulated	miR-423-5p ↓	Promote growth and metastasis in prostate cancer	[134]
Prostate cancer	Human prostate cancer cell lines DU145	Upregulated	miR-320b ↓	Promote cell cycle progression	[137]
Prostate cancer	Prostate cancer cell lines and mice	Upregulated	MYBL2/mTOR Axis ↑	Regulates glucose metabolism in prostate cancer	[136]
Prostate cancer	Prostate cancer cell lines and patient samples	Upregulated	EZH2 ↑	Induces oncogenic activities of EZH2	[231]
Breast cancer	Bioinformatic analyses	Upregulated	miR-497-5p ↓ SHOC2 axis ↑	Regulates the resistance of breast cancer cells to paclitaxel	[179]
Breast cancer	Patient tissue samples and cell line	Upregulated	microRNA-561-3p ↓ TOP2A ↑	Promote breast cancer progression	[23]
Breast cancer	Patient tissue samples and cell line	Upregulated	miR-339-5p ↓	Promotes poor prognosis in breast cancer	[232]
Cervical cancer	HeLa cells	Upregulated	IL-6/STAT3 ↑	HPV18 E6/E7-mediates MALAT-1 upregulation to cervical cancer development	[167]
Cervical cancer	Cervical cancer tissue and cell lines	Upregulated	miR-485-5p ↓ MAT2A ↑	Promotes the proliferation of cervical cancer cells	[171]
Cervical cancer	Cervical cancer tissue and cell lines	Upregulated	miR-370-3p ↓ PI3K/Akt ↑	Promote the cisplatin resistance of cervical cancer cells	[233]
Cervical cancer	Cervical cancer tissue, cell line, and nude mice	Upregulated	miR-124 ↓	Promotes the proliferation of cervical cancer cells	[174]
GBM	GBM tissues and cell lines	Upregulated	miR-199a ↓ ZHX1↑	Promotes GBM development and progression	[234]
GBM	GBM cell lines and mice	Upregulated	MDR-associated proteins ↑ (MDR1, MRP5 and LRP1) and EMT related proteins (ZEB1, Snail and SLUG)	Promote GBM cell resistance to TMZ	[235]
GBM	GBM cell lines nude mice, and patient tissue samples	Upregulated	NF-kB ↑ p53 ↑	MALAT-1 as a target for chemosensitization of GBM	[236]
Lung cancer	Peripheral blood mononuclear cell	Downregulated	MDSCs ↑	MALAT-1 negatively regulates MDSCs	[214]
Lung cancer	A549 and HCC 1299 human lung adenocarcinoma cell lines		miR-200a ↓	Promotes lung cancer development and gefitinib resistance	[218]
NSCLC	Tumor tissues and adjacent normal tissues and lung cancer cell lines	Upregulated	miR-185-5p ↓ MDM4 axis ↑	Promote NSCLC progression	[22]
NSCLC	NSCLC cell lines and nude mice	Upregulated	miR-374b-5p ↓ SRSF7 ↑	Promote NSCLC progression	[237]
NSCLC	Tumor tissues, adjacent normal tissues, and NSCLC cell lines	Upregulated	miR-202 ↓	Promotes growth and invasion of NSCLC	[191]
Ovarian cancer	Epithelial ovarian cancer tissues and normal ovarian tissues	Upregulated	PI3K-AKT ↑	Promotes ovarian cancer development and metastasis	[238]
Ovarian cancer	Tumor tissue samples and cell lines	Upregulated	cyclin D1, p-PI3K, and *p*-Akt, ZEB2 and YAP ↑	Promotes ovarian cancer development	[239]
Ovarian cancer	Tumor tissue samples and cell line	Upregulated	MMP13 ↓ MMP19 and ADAMTS1↑	Induces ovarian cancer cell proliferation and migration	[152]
Laryngeal squamous cell carcinoma	TCGA database, LSCC specimens and adjacent normal counterparts, and HepG2 cell.	Upregulated	miR-362-3p ↓	Promotes the proliferation and invasiveness of laryngeal squamous cell carcinoma	[240]
Osteosarcoma	Osteosarcoma tissues and matched adjacent healthy tissues and cell lines	Upregulated	miR-590-3p ↑	Promotes osteosarcoma cell growth, invasion, and migration	[241]
Osteosarcoma		Upregulated	miR-873-5p ↓ ROCK1 ↑	Modulate the growth, invasion apoptosis, and migration of osteosarcoma Cells	[242]
Osteosarcoma	Tumor tissue samples and MG63 cell	Upregulated	TGIF2b ↑ miR-129 ↓	Promotes growth, invasion, and migration of osteosarcoma Cells	[243]
Osteosarcoma	Osteosarcoma cell lines and Zebrafish	Upregulated	miRNA-150-5p ↓ VEGFA ↑	Promotes angiogenesis in osteosarcoma microenvironment.	[244]
Osteosarcoma	Osteosarcoma cell lines and nude mice	Upregulated	β-catenin ↑ E-cadherin ↓	Promotes osteosarcoma progression	[245]
Pancreatic cancer	TCGA databases, bioinformatic analysis, and pancreatic cancer cell lines	Upregulated	PD-L1 ↑	MALAT-1 is involved in METTL3-mediated promotion of PD-L1 expression in pancreatic cancer.	[246]
Pancreatic cancer	Pancreatic cancer tissues and corresponding normal tissues, and normal human pancreatic duct epithelial cell line	Upregulated	p53 ↓ MiR-129-5p ↓	Regulate pancreatic cancer progression	[247]
Pancreatic cancer	Human tissue samples and cell lines	Upregulated	miR-217 ↓	Regulate KRAS expression in pancreatic cancer	[248]
Pancreatic cancer	Pancreatic cancer specimens and Bxpc-3 and Panc-1 and human pancreatic duct epithelial cells	Upregulated	HuR-TIA-1 ↓	Promotes malignant pancreatic cancer proliferation	[115]

lncRNAs, an array of RNA molecules with over 200 bases, have no open reading frames (ORFs) and cannot code for proteins [7]. These lncRNAs have been shown to be linked to many pathogenic processes, such as growth, invasion, and apoptosis [8]. Additionally, overexpression of several lncRNAs has been linked to reduced disease-free survival and survival rates in patients with cancer, underscoring the possibilities as targets for treatment and diagnostic and prognostic indicators [9,10]. Ji and colleagues first identified MALAT-1 as an 8.5-kilobase lncRNA in 11q13 [11]. Recent studies have shown that MALAT-1 plays a vital role in the onset and spread of cancer. It achieves this gaol by modifying various molecular signaling pathways, including phosphatidylinositol 3-kinase (PI3K)/protein kinase B (Akt), WNT/β-catenin, Nuclear factor kappa B (NF-κB), and mitogen-activated protein kinase (MAPK)/extracellular signal-regulated kinase (ERK), resulting in alterations in tumorigenicity, invasion, cell cycle, immunity, migration, and angiogenesis [[12], [13], [14], [15], [16]].

Furthermore, MALAT-1 is linked to clinicopathological characteristics, including tumor size, location, differentiation, stage, and treatment resistance. In addition, increasing data points to the potential of abnormal MALAT-1 expression as a biomarker for tumor diagnosis and prognosis in tumor tissues and/or bodily fluids [17]. This study offers a comprehensive examination of the pathogenic molecular function of MALAT-1 in cancer, focusing on its role in promoting carcinogenesis and the development of chemoresistance. We will also discuss the diagnostic and therapeutic applications of MALAT-1 in cancer therapy. By consolidating the current knowledge on MALAT-1, this review aims to enhance our understanding of its pathogenic molecular role and provide insights into potential avenues for future research and therapeutic interventions. Overall, unraveling the complex molecular mechanisms underlying the pathogenic role of MALAT-1 in cancer will deepen our understanding of tumor biology and hold great promise for developing novel diagnostic tools and therapeutic strategies. Targeting MALAT-1-mediated processes may pave the way for more effective personalized treatments and improved clinical outcomes in cancer patients.

## 2 The summary of the multifaceted functions of MALAT-1 in cancer pathophysiology

The idea that cancer shows particular features during its growth and progression has been supported by several research investigations throughout the years, such as boosted cell growth, escape of cell death, promotion of angiogenesis, induction of metastasis, escape of immune monitoring, and raised resistance to chemotherapy [18,19]. MALAT-1 has been identified as a crucial regulator in cancer pathophysiology among the multiple molecular actors participating in these mechanisms.

## 3 MALAT-1 and cancer cell growth, metastasis, and invasion

The growing body of evidence revealed the significant role of MALAT-1 in cancer pathophysiology, including cancer cell growth, metastasis, and invasion [[20], [21], [22], [23], [24], [25]]. For example, MALAT-1 has been discovered to affect cell growth in several cancer types. For instance, MALAT-1 stimulates tumor development in retinoblastoma by blocking miR-124, which causes Slug, a crucial element of the MAPK/ERK pathway, to be upregulated. This activates MAPK/ERK signaling and aids in tumor propagation [26]. A complicated set of molecular processes is involved in metastasis, a significant contributor to cancer-related fatalities. EMT, a crucial stage in the metastatic process, is the main way MALAT-1 affects cancer propagation [27,28]. In this regard, MALAT-1 is a cancer-causing gene in ovarian cancer, and its blockade significantly hinders EMT. As a result, the mesenchymal markers (*N*-cadherin, vimentin, and snail) and matrix metalloproteinases (MMPs) are less expressed, accompanied by increased expression of the epithelial marker E-cadherin. Additionally, it has been discovered that MALAT-1-mediated control of EMT in ovarian cancer cells involves the PI3K/AKT signaling pathway [29]. Recently, Duan et al. investigated the role of MALAT-1 in the growth and metastasis of head and neck squamous cell carcinoma (HNSCC) and uncovered a novel mechanism underlying its oncogenic functions [20]. They found that MALAT-1 expression was significantly upregulated in HNSCC tissues compared to normal squamous epithelium. Notably, higher MALAT-1 expression is observed in poorly differentiated tumors and those with lymph node metastasis [20]. Their in vitro and *in vivo* experiments demonstrated that targeting MALAT-1 significantly impairs the proliferative and metastatic capacities of HNSCC cells [20]. This implies that MALAT-1 plays a crucial role in promoting both the growth and metastasis of HNSCC. Mechanistically, they demonstrated that MALAT-1 exerts its oncogenic effects by inhibiting the von Hippel-Lindau tumor suppressor (VHL) through a non-canonical function of the Enhancer of Zeste 2 Polycomb Repressive Complex 2 Subunit (EZH2) [20]. Also, they showed that activating the EZH2/STAT3/Akt axis by MALAT-1 leads to the inhibition of VHL, stabilizing and activating β-catenin and NF-κB [20]. All in all, they revealed that the activation of these signaling pathways is implicated in promoting HNSCC growth and metastasis, providing mechanistic insights into how MALAT-1 contributes to the malignant progression of the disease.

## 4 MALAT-1 and cancer cell angiogenesis

Angiogenesis, which is the construction of new blood vessels, is essential for the progression of cancer because it makes it possible for tumors to get nutrition, growth stimulants, and oxygen, as well as spread to other organs [30]. A crucial component of cancer therapy is the suppression of angiogenesis, which aims to cut off the blood supply to tumor micro-regions and cause necrosis in solid tumor tissues. According to studies, MALAT-1 controls genes related to the cell cycle in endothelial cells, which regulates angiogenesis [[31], [32], [33]]. For example, Vimalraj et al. focused on understanding the role of MALAT-1 in osteosarcoma (OS)-induced angiogenesis [33]. The expression of MALAT-1 in human OS cells is significantly higher than in normal osteoblasts. This observation indicates that MALAT-1 may be dysregulated in OS, suggesting a potential role in the disease [33]. Their functional analyses, both in vitro and *in vivo*, demonstrate that MALAT-1 enhances OS-induced angiogenesis [33]. Mechanistically, their findings showed that silencing MALAT-1 leads to a downregulation of vascular endothelial growth factor A (VEGFA) expression in OS cells [33]. Upon further investigation, it is revealed that MALAT-1 selectively targets miR-150-5p and miR-150-5p, which, in turn, specifically targets VEGFA in OS cells [33]. Also, they demonstrated that MALAT-1 induces angiogenesis in the OS microenvironment by upregulating the expression of VEGFA by targeting miR-150-5p [33]. This intricate regulatory network involving MALAT-1, miR-150-5p, and VEGFA suggests a key role for MALAT-1 in modulating the angiogenic microenvironment in OS. The identified molecular mechanisms provide a potential target for therapeutic interventions to mitigate osteosarcoma's angiogenic potential.

## 5 MALAT-1 and cancer cell apoptosis

Apoptosis serves as the inherent mechanism for programmed cell death within the cell. Its significance is particularly pronounced in long-lived mammals, as it plays a pivotal role in developing and maintaining homeostasis [34,35]. When apoptotic control is compromised, cancer cells gain the ability to prolong their survival, allowing sufficient time for the accumulation of mutations. This, in turn, can increase invasiveness during tumor progression, stimulate angiogenesis, disrupt cell proliferation, and hinder the process of differentiation [35]. There are multiple mechanisms by which cancer cells may resist apoptosis: caspase function can be suppressed, or the stimulus for apoptosis can be deactivated [36]. Recently, it has been shown that MALAT-1 has a crucial role in cancer cell apoptosis [[37], [38], [39], [40]]. For instance, Peng et al. demonstrated in their study that downregulation of MALAT-1 leads to increased hepatocellular carcinoma (HCC) apoptosis and autophagy [40]. This suggests that MALAT-1 exerts anti-apoptotic and anti-autophagic effects, contributing to the survival and evasion of programmed cell death in HCC cells. Also, their findings revealed that downregulation of MALAT-1 promotes apoptosis and autophagy in HCC cells, and these effects are reversed by inhibiting miR-146a, indicating that miR-146a is crucial for mediating the downstream effects of MALAT-1 [40]. Peng et al. found that miR-146a directly targets the 3′-untranslated region of PI3K, a key component of the PI3K/Akt/mammalian target of the rapamycin (mTOR) signaling pathway [40]. They indicated that transfection with miR-146a mimic results in decreased PI3K protein levels, indicating a direct regulatory interaction between miR-146a and PI3K [40]. In conclusion, the study provides insights into the role of MALAT-1 in HCC development, highlighting its impact on cell proliferation, apoptosis, and autophagy through the regulation of the miR-146a/PI3K/Akt/mTOR signaling axis. The identified molecular interactions offer a potential avenue for therapeutic intervention in HCC.

## 6 MALAT-1 and cancer immunomodulation

The host's immune system is crucial in detecting and keeping track of tumor cells [41]. Since cancer is an intricate disease, it affects the immune system in a wide variety of ways and alters the immune system's structure and function. Complex relationships between distinct cell lineages in many organs control immune responses. Therefore, it is crucial to consider the broader immune landscape and the tumor microenvironment (TME) to improve their knowledge of tumor immunology [42]. The relationship between MALAT-1 and tumor immunity has been the topic of several recent investigations [14,43]. The investigation has shown that tumor tissues and tumor-associated macrophages (TAMs) exhibit higher quantities of MALAT-1 and fibroblast growth factor 2 (FGF-2) than normal tissue. The synthesis of inflammatory cytokines is seen to be inhibited by the MALAT-1-mediated release of FGF2 from TAMs, but tumor cell growth, migration, invasion, and the development of angiogenesis are promoted [43]. Additionally, it was previously shown that MALAT-1 interacts with NF-kB in the nucleus of lipopolysaccharide-activated macrophages, preventing NF-kB from attaching to the promoters of pro-inflammatory cytokine genes and decreasing the synthesis of tumor necrosis factor-α (TNF-α) and interleukin-6 (IL-6) [14]. A recent study by Y. Mekky et al. revealed that MALAT-1 plays a role in dampening innate and adaptive immune responses. This suppression occurs, in part, by targeting the miR-34a/MICA/B and miR-175p/PD-L1/B7–H4 axes in patients with triple-negative breast cancer (TNBC) and in cell lines [44]. Moreover, the cytotoxic effect of co-cultured natural killer (NK) cells and CD8^+^ cells is heightened when co-cultured with MDA-MB-231 cells transfected with MALAT-1 siRNA [44]. A study performed by Mekky et al. showed that MALAT-1 knockdown leads to the repression of programmed cell death ligand-1 (PD-L1) and B7–H4 expression levels [44]. Additionally, the ectopic expression of miR-17-5p in MDA-MB-231 cells markedly suppresses the expression of immunological checkpoint markers PD-L1 and B7–H4 [44]. Therefore, this study, by examining the miR-34a/MICA/B and miR-175p/PD-L1/B7–H4 axes in TNBC patients and cell lines, demonstrated that MALAT-1 plays a partial role in mediating innate and adaptive immune suppression processes [44].

## 7 The mechanistic role of MALAT-1 in various types of cancer

In this section, we will explore the mechanistic role of MALAT-1 in various types of cancer, including gastrointestinal cancers, reproductive system cancers, and lung cancer. It's important to note that the specific mechanistic roles of MALAT-1 can vary among different cancer types and even within subtypes of a particular cancer. Ongoing research continues to uncover the intricate details of how MALAT-1 contributes to cancer biology, and potential therapeutic strategies targeting MALAT-1 are being explored.

## 8 Gastrointestinal cancer

### 8.1 HCC

The prevalence of HCC is most pronounced in East Asia and Africa, but its occurrence and fatality rates are increasingly on the rise in the United States and Europe [45]. However, the precise molecular processes underpinning the emergence of HCC remain poorly understood. Increasing knowledge of these pathways is essential to enhancing diagnosis and therapy [46,47]. Recent research has identified lncRNAs as critical regulators of the tumorigenic process [48]. MALAT-1, one of these lncRNAs, has been linked to many cancers, such as breast, lung, and HCC [24,49,50].

Previous research has demonstrated that MALAT-1 dysregulation is connected to the development of HCC [51,52]. Molecular mechanisms underpinning the function of MALAT-1 in the development of HCC were studied recently by Malakar and colleagues [53]. They found that increased expression of MALAT-1 induces the production of the oncogenic Serine/Arginine-Rich Splicing Factor 1 (SRSF1), which functions as an oncogenic trigger in HCC [53]. They discovered that MALAT-1 expression was elevated in HCC tumor tissues and that MALAT-1 knockdown inhibited HCC cell growth, invasion, and production of pro-inflammatory mediators [54]. It was shown that MALAT-1 functions by attracting Brahma-related gene 1 (BRG1), a catalytic member of the chromatin remodeling complex SWItch/Sucrose Non-Fermentable (SWI/SNF), to the promoter region of the genes associated with inflammation (IL-6 and CXCL8), boosting NF-κB-mediated production of these components [54]. These results show that MALAT-1 boosts HCC growth by attaching to BRG1 and, via epigenetic processes, increasing the level of inflammation in HCC tissues. Thus, inhibiting MALAT-1 might be a possible treatment for HCC.

The study by Zhao et al. examined the function of MALAT-1 in hypoxic HCC cells and investigated its underpinning regulatory mechanism [55]. In hypoxia-stressed HCC cell lines (Huh7, SNU-423, PLC, and Hep3B), the research found that MALAT-1 was elevated, whereas miR-200a was negatively regulated [55]. MiR-200a is possibly implicated in the development of hypoxic HCC cancer since Zhao et al. discovered that miR-200a was considerably decreased in hypoxic HCC cells [55]. They found a relationship between miR-200a and MALAT-1, with MALAT-1 modulating the expression of miR-200a negatively in Hep3B cells [55]. Furthermore, in hypoxia-challenged Hep3B cells, overexpression of miR-200a reduced cell growth, migration, and invasion and triggered death, indicating a role in developing hypoxia-driven HCC. Besides, miR-200a mimic transfection and MALAT-1 knockdown had the same impact on Hep3B cells. These findings offer new light on the critical function of the lncRNA-miRNA regulatory network in cancer progression, especially in hypoxia-challenged HCC.

Liu et al. looked at how MALAT-1 controls the growth and dissemination of HCC [56]. They explored the role of MALAT-1 lncRNA in HCC and found that, in contrast to healthy liver controls, it was highly expressed in HCC tumor tissues. They discovered the function of MALAT-1 in increasing the migration and proliferation potential of HCC cells by knockdown or overexpression experiments and demonstrated its oncogenic property in this type of cancer [56]. They postulated that miR-125a-3p was responsible for mediating the MALAT-1-dependent control of forkhead box protein M1 (FOXM1) levels based on the function of MALAT-1 in regulating FOXM1 [56]. Through tests, it was shown that blocking miR-125a-3p reversed the alterations in FOXM1 levels brought on by MALAT-1 knockdown. The impacts of MALAT-1 knockdown on HCC cell migration and proliferation were likewise reversed by miR-125a-3p blocking, indicating that MALAT-1 controls these activities via miR-125a-3p [56]. These results suggest that MALAT-1 regulates FOXM1 expression by functioning as a miR-125a-3p sponge, accelerating the development of HCC.

Hou et al. investigated how miR-140 and MALAT-1 interact in regulating angiogenesis and immunosuppression characteristics [49]. Their research showed that MALAT-1 was required for the mechanisms of HCC metastasis known as revascularization in human umbilical vein endothelial cells (HUVECs) and M2 polarization of macrophages. Their findings showed that miR-140, a potent blocker of angiogenesis and M2 polarization, acts as a sponge for MALAT-1, which enables it to carry out its essential role. This new method highlights the intricate regulatory system responsible for HCC metastasis [49]. The significance of VEGF in angiogenesis has been proven via extensive study [57]. According to Hou et al., the knockdown of MALAT-1 decreased the amount of VEGF-A present in the conditioned media and inhibited VEGF-A production in HCC cells. When HUVECs were exposed to conditioned media from MALAT-1-silenced HCC cells, their migration and tube formation were hampered [49]. They also found that MALAT-1 and miR-140 interacted, with MALAT-1 acting as a sponge for miR-140. In HCC cells, miR-140 expression was increased due to MALAT-1 knockdown. According to growing evidence, miR-140-5p may target VEGF-A to control tumor angiogenesis [58,59]. Hou et al. showed that miR-140 inhibits the translation of VEGF-A mRNA by binding to the 3′-UTR of VEGF-A mRNA [49]. Therefore, focusing on MALAT-1 or miR-140 might slow the spread of HCC in the future.

Peng et al. examined how MALAT-1 influences the progression of HCC [40]. In two HCC cell lines and human HCC tissues, the research found that MALAT-1 was upregulated. Further investigation into the function of MALAT-1 in controlling the growth and survival of HCC cells revealed that silencing MALAT-1 decreased the proliferation and survival of HCC cells by inducing apoptosis and autophagy [40]. Previous research has connected miR-146a decreased levels to HCC [60], while miR-146a upregulation has been demonstrated to prevent HCC cell proliferation and invasion [61]. A study conducted by Peng et al. demonstrated a positive correlation between decreased expression of miR-146a and increased expression of PI3K in HCC cells. Furthermore, the study established a relationship between miR-146a and MALAT-1-mediated apoptosis and autophagy. These discoveries provide insight into the intracellular signaling networks of miR-146a [40]. The current work is the first to investigate the connection between HCC, MALAT-1, and miR-146a [40], despite a prior examination [62] showing that MALAT-1 downregulates miR-146b-5p in HCC. The PI3K/Akt signaling pathway is well recognized for its involvement in developing numerous malignancies, including HCC [[62], [63], [64]]. A study by Peng et al. reveals that miR-146a exerts a negative regulatory effect on the PI3K/Akt/mTOR pathway in HCC cell lines. This is achieved through the inhibition of PI3K, Akt, and mTOR expression, as well as the phosphorylation of Akt [40]. Overall, this research suggests that MALAT-1 may control the course of HCC by modulating HCC cell growth, autophagy, apoptosis, and miR-146a sponging.

Researchers used bioinformatics to find a target relationship between lncRNA MALAT-1 and miRNA-613 in HCC patient tissues [65]. According to the findings of Zhou et al., HCC patient tissues exhibit elevated levels of MALAT-1 lncRNA expression. The expression of miRNA-613 was markedly upregulated, and the capacity of liver cancer cells to invade was dramatically reduced in liver cancer cells with lncRNA MALAT-1 knockdown [65]. The observed enhancement in the invasive potential of hepatoma cells upon inhibition of miRNA-613 implies a potential role of lncRNA MALAT-1 in suppressing the expression of miRNA-613. This suppression may promote the dissemination and invasion of liver cancer cells through peripheral blood vessels [65]. An earlier investigation indicated significantly decreased levels of miR-613 in HCC [66], and a subsequent study by Wang et al. displayed significant downregulation of miR-613 in HCC [66,67]. By inhibiting miRNA-613 expression in HCC cells, Zhou et al. looked into the possible contribution of lncRNA MALAT-1 to promote HCC metastasis via peripheral vascular infiltration. They used the Transwell test to measure the invasiveness of HCC cells and shut down the lncRNA MALAT-1 to investigate this. The research showed that suppressing miRNA-613 expression after lncRNA MALAT-1 was knocked down boosted the invasion of HCC cells. All in all, the research on the function of MALAT-1 in HCC emphasizes the multiple roles it plays in promoting cell proliferation, suppressing apoptosis, promoting metastases, and inducing treatment resistance. The possible use of MALAT-1 as a target for therapy for creating cutting-edge methods to enhance the treatment and management results of HCC may be better understood through comprehension of these molecular connections and pathways. Fig. 2 illustrates the multifaceted impact of MALAT-1 on HCC progression and metastasis.

**Fig. 1:**
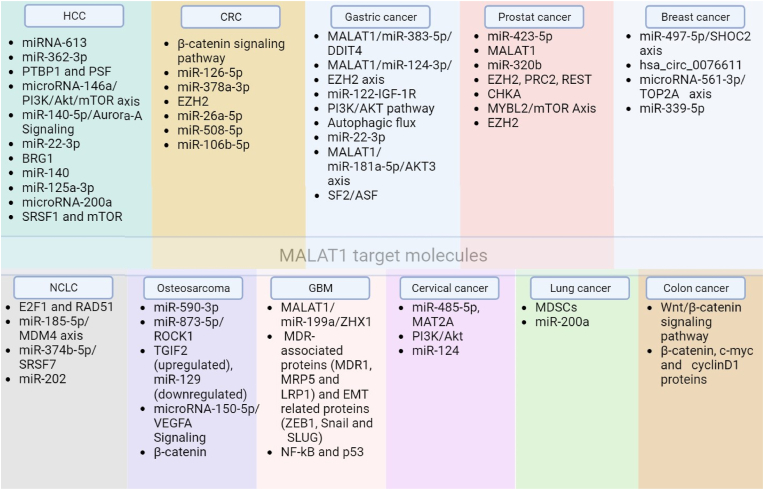
Fig. 1 provides a concise overview of the mechanisms and targets of MALAT-1 that play a role in the pathogenesis of various cancers.

**Fig. 2:**
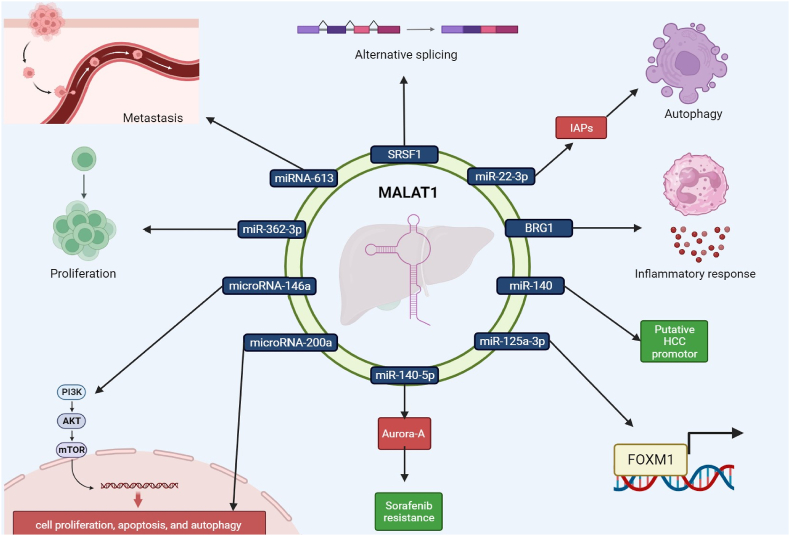
This figure illustrates the multifaceted impact of the long non-coding RNA MALAT-1 on hepatocellular carcinoma (HCC) progression and metastasis. MALAT-1 exerts its influence through various molecular interactions involving key regulators and signaling pathways. For example, MALAT-1 modulates HCC cell proliferation, apoptosis, and autophagy via sponging miR-146a, which regulates HCC progression.

### 8.2 Colorectal cancer (CRC)

One of the main factors contributing to cancer-related mortality globally is CRC. In order to develop precise therapeutic interventions and optimize patient outcomes, it is imperative to gain a comprehensive understanding of the molecular mechanisms underlying the initiation and progression of the condition [68,69]. In recent years, lncRNAs have become essential participants in many biological processes, including the emergence of cancer. MALAT-1 is one such lncRNA that has attracted much interest in CRC [70,71]. Li et al. have concentrated on how MALAT-1 affects oxaliplatin-induced metastasis and chemoresistance in CRC patients [72]. According to the research, patients with advanced CRC who had high expression of MALAT-1 had lower patient survival rates and worse outcomes with oxaliplatin-based chemotherapy [72]. Li et al. conducted tests to find out how suppressing MALAT-1 expression in CRC cells affected the cells' behavior. They discovered that MALAT-1 knockdown increased the expression of E-cadherin, a protein linked to preserving the epithelial phenotype, and prevented the formation of EMT in response to oxaliplatin. This implies that suppressing EMT by targeting MALAT-1 may help prevent oxaliplatin-induced chemoresistance [72]. The scientists discovered that oxaliplatin-induced EMT and chemoresistance in CRC cells may be reversed by specifically inhibiting either MALAT-1 or EZH2 [72]. The research also found that MALAT-1 and miR-218, another regulatory RNA molecule, interacted. This interplay additionally demonstrated MALAT-1's prognostic significance in patients receiving conventional FOLFOX therapy [72]. Overall, this research showed that targeting MALAT-1 or its relationship to EZH2 may provide novel approaches to increase the efficacy of chemotherapy based on oxaliplatin in treating CRC.

Sun and colleagues explored how YAP1 affected CRC and the regulatory system, including the YAP1-MALAT-1-miR-126-5p axis [73]. A potent oncogene that is increased in most malignancies is YAP1 [74,75]. YAP1 increased colon cancer cell growth, invasion, and motility in *in vitro* and *in vivo* tests, demonstrating its function in promoting aggressiveness in CRC [73]. A total of 11 lncRNAs exhibited alterations in their expression levels after transfection into HCT116 cells using either siYAP1 or control siRNA. Notably, the expression of MALAT-1 was significantly affected. Further investigation, employing bioinformatics prediction, a dual luciferase assay, RNA immunoprecipitation (RNA-IP), and an RNA pull-down assay, uncovered that the activation of yes-associated protein 1 (YAP1) resulted in the upregulation of metastasis-associated molecules, particularly VEGFA, by sequestering miR-126-5p in CRC [73]. The MALAT-1-miR-126-5p axis, which is YAP1-induced, was identified as an essential modulator of angiogenesis and EMT in CRC. The connection of YAP1 with poor overall survival and the development of malignant behaviors indicated its oncogenic involvement in CRC. The YAP1-MALAT-1-miR-126-5p axis seems to regulate critical CRC progression mechanisms, offering novel biomarkers and targeted therapies.

MALAT-1 expression in colon cancer tissues, its impact on SW480 cells' proliferation and apoptosis, and the signaling route underlying these effects were all examined by Zhang and colleagues [38]. They discovered that the expression of MALAT-1 was much more significant in colon cancer tissues when compared with para-carcinoma tissues, indicating that it might be involved in the emergence of colon cancer [38]. In several different kinds of tumors and disease models, the Wnt/β-catenin signaling pathway has been associated with death and cell growth; it may even have the opposite regulatory impact [76,77]. Apoptosis-related proteins were evaluated by Zhang et al. in SW480 cells that had been transfected with MALAT-1 siRNA. Moreover, MALAT-1 siRNA-transfected SW480 cells showed a spike in the ratio of cleaved caspase-3 to truncated caspase-3. The research showed that MALAT-1 siRNA-transfected SW480 cells decreased the production of Wnt and β-catenin. This suggested that MALAT-1 might alter the activity of the Wnt/β-catenin signaling pathway, which might affect cell proliferation and death [38].

In a different study, Zhang and colleagues explored the regulatory mechanism behind MALAT-1 and looked into its expression and function in CRC [78]. According to the study conducted by Zhang and colleagues, MALAT-1 expression was elevated in CRC patients, and it exhibited correlations with a poorer prognosis, lymph node metastases, and an advanced TNM stage. Inhibiting MALAT-1 resulted in a reduction of CRC cell proliferation and invasion in vitro [78]. They thus proposed that MALAT-1 could promote the development of CRC malignancy, which aligns with earlier research. The functional testing results demonstrated that suppressing MALAT-1 expression reduced CRC cells' proliferative and invasive capabilities in vitro. This indicates that MALAT-1 promotes CRC cell growth and invasion, supporting its function in the development of CRC [78]. According to earlier research, miR-508-5p functions as a tumor suppressor in numerous cancers [79,80]. According to the study, MALAT-1 may act as a competing endogenous RNA (ceRNA) for miR-508-5p in the development of CRC [78].

Autophagy regulation in the growth of CRC has been connected to bone morphogenetic protein (BMP)-Suppressor of Mothers Against Decapentaplegic (SMAD) signaling [81]. By increasing the expression of the autophagy-related gene 5 (ATG5), Zhou et al. study the function of Smad1, a significant effector of BMP2-Smad signaling, in triggering autophagy [71]. The research also investigated the role of MALAT-1 in regulating Smad1 expression via miR-26a-5p and its effects on the development of CRC [71]. MiR-26a plays a significant role in illnesses, including cancer, and Smad1 has long been identified as one of its primary target genes [82,83]. Numerous studies on colorectal cancer have demonstrated that miR-26a plays a crucial regulatory role in oncogenesis and metabolism [[84], [85], [86], [87]]. The researchers discovered that Smad1, a part of BMP2-Smad signaling, promotes ATG5 expression to cause autophagy. The proximal promoter region of ATG5 is bound by Smad1, resulting in the activation of gene expression. Furthermore, it has been demonstrated that BMP2 can induce autophagy in CRC [71]. Additionally, they discovered that ATG5 knockdown partly reversed the enhancement of CRC cell proliferation and migration caused by Smad1. This shows that ATG5 is implicated in how Smad1 affects the development of CRCs [71]. Additionally, the outcomes of their investigation demonstrated that MALAT-1 functions as a ceRNA that competes with miR-26a-5p for binding, controlling the de-repression of its downstream target, Smad1. MALAT-1 indirectly impacts Smad1 expression by encircling miR-26a-5p [71]. Finally, a clinical study demonstrated that Smad1 expression was negatively linked with miR-26a-5p and favorably correlated with MALAT-1 in CRC samples, further demonstrating their regulatory connections [71]. The regulatory mechanisms of autophagy in the development of CRC are clarified by this work. Smad1, a crucial player in BMP2-Smad signaling, promotes ATG5 expression to cause autophagy. Additionally, Smad1 upregulation facilitates the growth and migration of CRC tumors.

In the setting of CRC carcinogenesis, Castellani et al. studied the biological and cellular processes impacted by miR-378a-3p [88]. They investigated its interactions with two lncRNAs—MALAT-1 and Nuclear Paraspeckle Assembly Transcript 1 (NEAT1)—to better understand its effects on CRC-SCs (CRC stem-like cells) [88]. MALAT-1 and NEAT1, markedly excessively expressed in various kinds of cancer, have been shown to be promoters of multiple biological processes. They play roles in transcription, alternative splicing, and/or miRNA sponging, leading to the upregulation of genes associated with cancer-related activities such as enhanced migration, blood vessel formation, cell growth, and invasion [89,90]. Additionally, it has been demonstrated that miR-378 controls apoptosis in breast cancer by communicating with GAS5 lncRNA, while it targets GAPLINC lncRNA to regulate the development and cell cycle progression of gastric cancer cells [91,92]. It was determined that the expression of miR-378a-3p was reinstated, resulting in a reduction of tumorigenicity in both CRC and CRC stem cell (CRC-SC) lines. Furthermore, miR-378a-3p restoration dramatically reduced tumor development in two CRC-SC xenograft animal models, indicating tumor-suppressive activity in CRC [88]. The study demonstrates that the expression of two lncRNAs, MALAT-1 and NEAT1, is regulated by miR-378a-3p [88]. MALAT-1 and NEAT1 expressions were shown to be inversely related to miR-378a-3p in patient-derived CRC-SC lines. This suggests that miR-378a-3p affects the amounts at which these lncRNAs are expressed [88]. This result is consistent with the hypothesis that miR-378a-3p interacts with and controls the expression of MALAT-1 and NEAT1 to function as a tumor suppressor in CRC tumorigenesis [88]. The tumor-suppressive function of miR-378a-3p in CRC, especially in CRC-SCs, is highlighted in this work. MiR-378a-3p is downregulated in CRC, which is linked to many tumorigenic pathways.

Wu and colleagues recently looked into the possibility that Jumonji domain 2 (JMJD2) might epigenetically control the promoter activity of MALAT-1 and the downstream β-catenin signaling pathway, consequently impacting the ability of CRC cells to spread [93]. JMJD2C was found to be expressed excessively in matched CRC tumor tissues from both primary and metastatic foci, according to the research. Additionally, CRC patients had a more favorable prognosis and a longer overall life when JMJD2C expression in original tumors was reduced [93]. Prior research suggested that MALAT-1 might facilitate CRC metastasis by controlling the β-catenin signaling pathway [94,95]. *In vitro* and *in vivo* CRC metastases were both increased by JMJD2C, according to the biological function study [93]. A molecular mechanism analysis revealed that JMJD2C translocated into the nucleus and decreased histone methylation levels at the H3K9me3 and H3K36me3 sites in the MALAT-1 promoter. In CRC cells, this epigenetic control increased MALAT-1 expression and improved the β-catenin signaling pathway [93]. The research showed that JMJD2C is essential for boosting CRC cells' capacity to spread both in vitro and *in vivo*. This impact is brought about by JMJD2C's epigenetic control of the histone methylation state at the MALAT-1 promoter, which causes MALAT-1 to be expressed more and the β-catenin signaling pathway to be more active.

### 8.3 Gastric cancer (GC)

Globally, stomach cancer, also known as GC, is a serious health hazard. It is the third most frequent cancer in the world and the fifth most prevalent cancer overall [96,97]. A group of proteins known as the serine- and arginine-rich (SR) family is essential for controlling alternative splicing. The most well-known member of the SR family, the pre-mRNA-splicing factor SF2/alternative splicing factor (SF2/ASF), is involved in alternative splicing and mRNA stability [98]. This molecule has been linked to several human malignancies, notably lung cancer and cervical cancer [99,100]. According to recent studies, one of MALAT-1's roles is to draw SR from the cytoplasm into the nucleus [101]. According to Wang et al.'s hypothesis, MALAT-1 prevents SF2/ASF from malfunctioning and preserves its stability, and its abnormal expression may be a significant GC process regulator. The scientists discovered that three separate GC cell lines (SGC-7901, MKN-45, and SUN-16) had much higher levels of MALAT-1 than healthy cells did. This abnormal amplification of MALAT-1 suggests that it may play a role in the initiation and spread of GC [102]. A possible relationship between MALAT-1 and SF2/ASF in the control of cellular processes was revealed by the intriguing finding that when MALAT-1 was reduced, the nuclear distribution and expression of SF2/ASF were significantly impacted [102]. Notably, the overexpression of SF2/ASF did not reverse the suppression of cell proliferation observed in cells with depleted levels of MALAT-1. This implies that supplementary mechanisms, apart from SF2/ASF, may exist that could account for the impact of MALAT-1 on cellular proliferation [102]. Overall, the work offers insightful information on how MALAT-1 operates in GC. It implies that MALAT-1 partially promotes GC cell growth via controlling SF2/ASF. These results raise the possibility of employing MALAT-1 as a biomarker for detecting GC and as a possible therapeutic target for its treatment. New approaches to treating this condition might be developed as a result of more studies in this area.

The function and underlying mechanism of MALAT-1 in GC were investigated by Lu et al. [103]. According to the research, MALAT-1 was substantially expressed in both gastric adenocarcinoma cell lines and the serum of individuals with the disease. This observation raises the possibility that MALAT-1 contributes to the initiation and development of gastric adenocarcinoma [103]. In the work of Lu et al., they discovered that MALAT-1 knockdown dramatically reduced cell proliferation and increased cell apoptosis, which was similar to the earlier findings [103]. Leukemia, HCC, and BC are only a few cancers to which miR-181a-5p has been linked [[104], [105], [106]]. The research clarified how MALAT-1 works by explaining its mode of action. Expression levels of the microRNA miR-181a-5p were shown to be lowered by MALAT-1. Because of this, the expression of RAC-serine/threonine-specific protein kinase (AKT3) is upregulated in response to reduced miR-181a-5p [103]. It seems that MALAT-1-induced reductions in miR-181a-5p levels, which result in enhanced AKT3 expression, encourage the development of GC cells. Using the AKT antagonist ipatasertib or overexpressing miR-181a-5p had comparable effects to MALAT-1 knockdown, highlighting the significance of the MALAT-1/miR-181a-5p/AKT3 axis in the development of gastric adenocarcinoma [103]. The MALAT-1/miR-181a-5p/AKT3 axis plays a critical role in the promotion of gastric adenocarcinoma, as shown by this research. AKT3 protein levels rise as a result of MALAT-1 and AKT3 competition for miR-181a-5p binding, which eventually aids gastric adenocarcinoma cell proliferation and survival. These discoveries provide insight into the molecular processes underlying the development of stomach adenocarcinomas and may provide possible therapeutic options for treating this deadly cancer type.

MALAT-1 has been associated with GC, and Zhu and collaborators investigated its potential as both a prognostic and diagnostic indicator [106]. Research findings indicate that individuals with GC had significantly elevated levels of MALAT-1 expression in their tumors as compared to adjacent healthy tissues. This implies that MALAT-1 may contribute to the initiation and spread of GC [106]. As shown here, MALAT-1 plays a cancer-causing function in GC by facilitating cancer cell proliferation, migration, and invasion. The PI3K-AKT pathway and lncRNA MALAT-1 have interacted in the past [107]. The research put out a putative mechanism for how MALAT-1 promotes the growth, migration, and invasion of GC cells. The PI3K/AKT pathway was discovered to be activated by MALAT-1. It is known that the PI3K/AKT pathway controls many biological functions, including the proliferation of cells, survival, and migration [106]. The primary finding of this study demonstrates a correlation between elevated levels of MALAT-1 in GC and enhanced invasion, cellular proliferation, and migration. Furthermore, it is suggested that this phenomenon may be associated with the activation of the PI3K/AKT pathway. Additionally, the expression of MALAT-1 in plasma shows promise as a predictive and diagnostic marker for GC. Comprehending the function of MALAT-1 in GC might lead to the discovery of new therapeutic targets and ultimately improve care for patients.

A study by Zhu et al. suggests that H2 can halt the spread of stomach cancer. There is a need for further investigation into how H2 affects the development of stomach cancer in animal models [108]. MALAT-1 modulated the expression of miR-30e/ATG5 to alter autophagy and apoptosis in SGC7901 GC cells and impart cisplatin resistance [109]. The direct binding and stabilization of Sox2 mRNA by MALAT-1 improved GC cells' stemness, chemo-, and radioresistance [110]. On the other hand, it increased miR-124-3p expression. The blocking effects of hydrogen gas on previously mentioned cellular activities were countered by the overexpression of the lncRNA MALAT-1 [108]. Additionally, the research discovered that miR-124-3p and the lncRNA MALAT-1 had a reciprocal regulatory connection wherein both suppressed the production of the other. Additional research using miR-124-3p mimics showed that they efficiently inhibited GC cell growth and migration and lncRNA MALAT-1-induced EZH2 expression [108]. The results of this research point to the possibility of using hydrogen gas as a therapeutic agent to treat stomach cancer. In-vivo tumor development was successfully prevented, and important cancer cell activities, including migration and proliferation, were decreased. The research also implicated MALAT-1/miR-124-3p/EZH2 in these effects. Future therapeutic strategies for the treatment of GC may be successful in targeting this axis. The work provides essential information on the molecular processes behind the impact of hydrogen gas on stomach cancer as well as its potential therapeutic significance.

### 8.4 Pancreatic cancer (PC)

PC is an invasive condition that is distinguished by a low five-year survival rate of less than 5 % and a median survival of just six months following diagnosis. Because of these factors, pancreatic cancer is the solid tumor with the lowest prognosis [111]. PC is a disease that affects the pancreas. This cancer is well recognized for having a high propensity to spread locally and to distant areas, and it often demonstrates medication resistance. However, the underlying biological processes that underlie these traits remain poorly known. According to recent studies, lncRNAs might be essential for the growth and spread of malignancies [112]. Notably, it has been shown that MALAT-1 is highly expressed in various solid tumors [51,102,113]. A study by Jiao et al. revealed a significant increase in the expression levels of the MALAT-1 gene in pancreatic cancer tissues compared to adjacent noncancerous tissues [114]. The study implies that MALAT-1, identified as an oncogenic lncRNA, may play a role in fostering malignancy in PC. This suggests that it could serve as a potential target for therapeutic intervention [114].

Li et al. researched the molecular mechanism behind the higher levels of MALAT-1 and its involvement in tumor virulence [115]. They found a positive linear connection between MALAT-1 and LC3B mRNA expression, a cellular autophagy marker. Modifying numerous molecules associated with cellular autophagic flux, such as LC3, P62, and LAMP-2, was another effect of MALAT-1 downregulation [115]. While LAMP-2 performs an essential role in decomposing the contents of autophagosomes by enabling the fusion of lysosomes with autophagosomes throughout the last stage of autophagic flux, P62 is known to function as a chaperone throughout autophagosome degrading. In the groups where MALAT-1 was silenced, the research discovered that P62 levels were noticeably higher, whereas LAMP-2 levels were lower. Additionally, MALAT-1 knockdown drastically decreased LC3 degradation and colocalization in pancreatic cancer cells. Their results show that MALAT-1 affects autophagosome destruction rather than autophagosome production or fusion with lysosomes [115]. Scientists found that HuR, an RNA-binding protein, and TIA-1 activity were both affected by MALAT-1, which induced autophagy [115]. Li et al. discovered the mechanism by which MALAT-1 interacts with HuR and that silencing MALAT-1 dramatically improves the posttranscriptional control of TIA-1, inhibiting autophagy even further. According to the researchers, MALAT-1 could control carcinogenesis by activating autophagy through HuR-TIA-1 [115].

Research by Lee et al. investigated how environmental toxins affected the expression of MALAT-1 in PC tissues and cells [116]. Environmental exposure is thought to be a significant risk factor for chronic pancreatic inflammation and cancer, according to growing epidemiological research. According to the study's findings, exposure to environmental toxins activates the aryl hydrocarbon receptor (AHR), promoting the development of MALAT-1 and strengthening the epigenetic silencing ability of EZH2 [116]. These results suggest a possible pro-oncogenic function for AHR in epigenetic dysregulation, given that elevated expression or impaired activity of EZH2 or MALAT-1 have been linked to cancer stem cell characteristics and malignancy [114,117]. AHR is a key molecule that connects environmental contact with EZH2-mediated epigenetic abnormalities in pancreatitis and PC via the activation of MALAT-1 [116]. The research results suggested that AHR antagonism may be a fresh preventative tactic since both AHR antagonists and depletion stopped the stimulation of MALAT-1 and the rise in EZH2 enzyme activities, both in vitro and *in vivo* [116]. These findings reveal a unique mechanism by which environmental exposure induces epigenetic changes in pancreatic tissue and cancer cells by activating the AHR-MALAT-1-EZH2 signaling axis.

The function and mechanism behind importin 7 (IPO7) action in PC were examined in a different study by Xu and colleagues [118]. IPO7 is commonly overexpressed in various malignancies and plays a role in the advancement of diverse cancer types [119]. Notably, it has been discovered that IPO7 depletion activates p53 and results in p53-dependent cell cycle arrest [120]. According to Xu and colleagues., PC tissues exhibited a significant upregulation of IPO7 expression, and its overexpression was linked to a reduced survival time for those with the disease [118]. Functional tests showed that IPO7 overexpression greatly aided PC cell growth, migration, invasion, and suppression of apoptosis, along with a reduction in p53 expression. On the other hand, IPO7 knockdown boosted p53 expression and reduced the aggressive properties of PC [118]. The research verified that IPO7 positively controls MALAT-1 in pancreatic cancer cells, indicating that the IPO7/p53/MALAT-1 axis is involved in the evolution of PC [118]. Xu and colleagues discovered a miR-129-5p response region in PC cells within the MALAT-1 sequence, demonstrating that MALAT-1 functions as a sponge to sequester and suppress miR-129-5p [118]. It's significant that IPO7 was discovered to be a new target gene of miR-129-5p and that it was negatively regulated by it. A positive feedback loop coupling IPO7/p53/MALAT-1/miR-129-5p was postulated as a contributor to PC advancement [118], considering that IPO7 positively controls MALAT-1. In conclusion, our results show that IPO7 is a new oncogene in PC and that the positive feedback mechanism between IPO7, p53, MALAT-1, and miR-129-5p promotes the development of this fatal condition.

The distinct TME of pancreatic cancer, which presents difficulties for immunotherapy, was examined in the current work by Song et al. [121]. PC has a distinct TME, which presents obstacles to immunotherapy and demands more investigation [122]. M6A methylation, one of the most frequent mRNA alterations, contributes to carcinogenesis [123]. They mainly found risk signs for GNL3, CAPRIN1, METTL3, YWHAG, ALKBH5, and PCIF1. Song et al. discovered a considerable rise in METTL3 expression in pancreatic cancer [121], which is consistent with a current investigation on PDAC cells [114]. These results highlighted the considerable m6A RNA methylation regulator expression variations between tumor and normal tissues [121]. Additionally, research points to the overexpression of METTL3 as a factor in PC development and invasion [124]. This is indicated by its involvement in boosting programmed death-ligand 1 (PD-L1) expression and worsening the malignant phenotype in oral squamous cell carcinoma [125]. It has been discovered that METTL3 regulates PD-L1 expression in a range of cancer types. In PC patients, Song and colleagues found a marginal correlation between METTL3 and PD-L1 expression, and they showed that METTL3 positively controls PD-L1 expression in PC cells [121]. Glioma development has been associated with increased stability of MALAT-1, which was made possible by METTL3 [126]. Song et al. discovered that METTL3 regulated the expression of MALAT-1 in PC cells [121]. Through many processes, including miRNA sponging and association with EZH2, MALAT-1 has been demonstrated to promote PD-L1 expression [127,128]. According to the information provided by Song and colleagues, MALAT-1 may control how much PD-L1 is expressed by PC cells [121]. These results suggest that METTL3 may target the lncRNA MALAT-1 in PC cells to control the expression of PD-L1. Further investigation is vital to thoroughly understand the complex molecular pathways behind MALAT-1's role in developing PC and to examine its therapeutic potential in treating this fatal condition.

## 9 Reproductive cancers

### 9.1 Prostate cancer (PCa)

One of the most common cancers impacting men globally is PCa [129,130]. Despite substantial advances in research and therapy, the molecular pathways underlying the onset and spread of PCa are still poorly understood [131]. By controlling several cellular processes, lncRNAs, such as PCa, have become important participants in cancer biology [131,132]. MALAT-1 is one such lncRNA that has attracted much interest in studying PCa [133,134]. In contrast to normal prostate tissues, prostate cancer tissues have been shown to express MALAT-1 abnormally. It has been linked to the development of cancer and negative patient outcomes. Multiple investigations have demonstrated a connection between increased MALAT-1 levels and tumor development, invasion, and metastasis in PCa. Further research into MALAT-1 is crucial since it has also been linked to promoting resistance to several anticancer treatments [[133], [134], [135], [136]].

In recent research, Dai et al. investigated the relevance of MALAT-1 in PCa, especially its function in controlling the cell cycle and androgen receptor (AR) signaling [137]. It has been demonstrated that androgen and AR signaling are essential for the growth of prostatic tissue as well as the progression of PCa [138,139]. In PCa cells subjected to androgen stimulation, MALAT-1 expression was shown to be increased concurrently with a rise in the expression of AR. This raises the possibility that MALAT-1 and AR signaling are related to PCa [137]. The investigators discovered a substantial decrease in dihydrotestosterone (DHT) administration-induced cell growth and cell cycle development in PCa cells after they silenced MALAT-1. This suggests that MALAT-1 is essential to modulating these mechanisms and might affect the development and spread of PCa [137]. The research showed that MALAT-1 interacts with miR-320b and inhibits its expression. As a result, MALAT-1 serves as a sponge for miR-320b in this instance, blocking it from concentrating on other transcripts [137]. The scientists discovered that AR is targeted explicitly by miR-320b in PCa cells. MiR-320b may modify AR signaling and alter the behavior of PCa cells by adversely modulating AR expression [137]. The consequences of MALAT-1 silencing, including the suppression of cell growth and development of the cell cycle, reverted when miR-320b was repressed or AR was upregulated. This supports the idea that the MALAT-1-miR-320b-AR axis is critical in controlling prostate cancer cell behavior [137]. These findings show the functional importance of MALAT-1 in PCa, particularly in regulating AR signaling and cell cycle progression.

Yadav and colleagues looked into the function of MALAT-1 in prostate cancer [133]. For metastatic castrate-resistant PCa (mCRPC), poly (ADP-ribose) polymerase inhibitors (PARPi) have demonstrated encouraging outcomes as targeted treatment approaches. Nevertheless, only a small proportion of individuals with defects in the homologous recombination (HR) pathway have benefited clinically from them. Understanding potential combination therapies is crucial for expanding the application of PARPi therapy to a broader range of patients [133]. The work reveals the pathway through which inhibiting MALAT-1 affects the course of the disease. EZH2, a component of the polycomb repressor complex-2 (PRC2), is decreased by MALAT-1 suppression. The important regulator of neuroendocrine development, RE1 Silencing Transcription Factor (REST), is upregulated in response to reduced EZH2 levels. The improvement of genomic integrity and the development of illness are both influenced by this molecular chain reaction. In PCa cells, silencing MALAT-1 changes the gene expression profile for HR. A BRCA-like phenotype may result from a dysfunctional HR system, which is a crucial DNA repair route [133]. BRCAness is a condition in which tumor cells have features that are comparable to those of cells with BRCA mutations. As a result, BRCAness makes tumor cells sensitive to treatment with PARP antagonists. The findings of this study indicate that patients who exhibit resistance to anti-androgens and conventional chemotherapeutic agents may potentially develop susceptibility to PARP inhibitors through the inhibition of MALAT-1 [133]. Investigators might boost the efficacy of PARPi therapy in a larger patient group by targeting MALAT-1. The discovery of the MALAT-1-EZH2-REST pathway also provides information on possible treatment targets to stop the course of the disease and prevent neuroendocrine differentiation in mCRPC.

In the setting of PCa, Ferri and colleagues [134] investigated how MALAT-1 interacts with miR-423-5p, a possible target indicated by in silico research. According to a bioinformatic study of data from the Cancer Genome Atlas (TCGA), MALAT-1 expression is associated with worse survival, metastatic frequency, and high Gleason grades in PCa patients. According to research by Ferri et al., poor differentiation grade and the existence of lymph nodes and distant metastases are both related to MALAT-1 expression levels that are much greater in cancer tissues than in normal tissues. As a result, they demonstrate for the first time that MALAT-1 may have diagnostic and prognostic significance in PCa. To emphasize prospective interventional techniques based on targeting MALAT-1 in PCa, they also investigated the potential mechanisms that could control the expression and activity of MALAT-1 in PCa [134]. The research found that miR-423-5p regulates MALAT-1 in PCa cells. Direct interaction between miR-423-5p and MALAT-1 causes MALAT-1 expression to be downregulated and its ability to promote invasion, migration, and proliferation to be inhibited. This demonstrates a unique regulatory mechanism that controls MALAT-1 function in PCa cells via a particular microRNA [134]. The researchers identified the downstream pathways impacted by miR-423-5p expression and MALAT-1 decreased levels through NanoString analysis. They discovered several changes in angiogenic and metastasis response-related genes. This suggests that miR-423-5p is essential for controlling critical signaling pathways linked to the development of PCa [134]. Increasing the expression of miR-423-5p improved survival and reduced the development of metastatic disease in a mouse xenograft model. This highlights the therapeutic advantages of focusing on miR-423-5p as a way to stop the spread and growth of PCa [134]. This work clarifies the significance of MALAT-1 in the origin and development of PCa tumors.

De Martino and colleagues look at how MALAT-1 regulates the production of choline kinase alpha (CHKA) and how this affects PCa metabolism, especially in connection to AR signaling [140]. By altering AR signaling, choline kinase (CHK) has previously been recognized as a viable target for regulating CRPC [141,142]. Targeting MALAT-1 in PCa cells resulted in a significant decline in the expression of CHKA, especially in cells with a high dose responsiveness to the AR and its variations. Additionally, MALAT-1 removal caused the concentrations of total choline-containing metabolites to drop, especially phosphocholine (PCho), suggesting modifications in PCa cell metabolism [140]. By lowering the expression levels of genes associated with lipid metabolism, such as HCC, MALAT-1 knockdown decreased glucose absorption and lipogenesis [143]. The research looked at the epigenetic processes that underlie CHKA control by MALAT-1. They discovered that critical AR binding sites are situated in the CHKA intron-2 region, where MALAT-1 deletion led to a significant rise in regulatory histone remodeling. This shows that MALAT-1 helps keep the CHKA gene locus's chromatin active, enabling optimal AR binding and transcriptional activation. The investigators also investigated the interaction between MALAT-1 and AR signaling. They observed that treating androgen-responsive PCa cells (LNCaP) with MALAT-1 targeting also blocked the MALAT-1-dependent silencing of CHKA. However, this impact was not seen in hormone-refractory cells (22RV1). This suggests that androgen receptor activation and CHKA regulation by MALAT-1 are closely related [140]. In conclusion, their work emphasizes the significance of MALAT-1 as a controller of CHKA expression and its function in the metabolic rewiring of prostate cancer cells.

Mu et al. looked into the role of MALAT-1 and its mechanisms in PCa onset and development. MALAT-1 was shown to be very important in controlling PCa cell growth and glucose metabolism [136]. MYBL2 promotes the malignant evolution of malignancies through cancer proliferation, treatment resistance, and metastasis [144,145] and is a possible oncogene and biomarker in invasive tumors [146]. Intriguingly, MALAT-1 controls SRSF1's interaction with MYBL2 exons in pre-mRNA [147]. Since suppression of MALAT-1 results in significant changes in the protein and mRNA levels of MYBL2, Mu et al. conclude that MALAT-1 and MYBL2 are mutually regulated in PCa cells [136]. It was discovered that MALAT-1 upregulates MYBL2 to increase the phosphorylation level of the mTOR pathway. The mTOR pathway is a crucial component of glucose metabolism and could serve as a target for therapy [148]. Additionally, Mu and colleagues investigated the significance of the MALAT-1/MYBL2/mTOR axis in glucose metabolism and discovered that this axis influences various glycolysis-related products in PCa cells [136]. The processes of glycolysis and lactate amounts in PCa cell lines were affected by inhibiting the MALAT-1/MYBL2/mTOR axis. This suggests that the MYBL2-mTOR axis is the pathway via which MALAT-1 regulates glucose metabolism [136]. In conclusion, evidence reveals the relevance of MALAT-1 in controlling prostate cancer glucose metabolism and progression. It emphasizes how crucial a role the MYBL2-mTOR axis plays as a mediator in this process.

### 9.2 Ovarian cancer (OvCa)

The most fatal gynecological disease in the US is OvCa [149]. Most OvCa cases are detected at an advanced stage (IIIc or above), and systemic chemotherapy is the usual course of treatment. Late-stage ovarian cancers are extremely resistant to primary therapy, with 80 % or more of cases relapsing after initial success [150,151]. MALAT-1 is found in abundance in OvCa tissues and cell lines, according to many investigations [[152], [153], [154]]. According to recent research by Sun et al., upregulation of MALAT-1 causes the JAK2/STAT3 signaling pathway to be activated, which causes OvCa cells to proliferate and suppress cell death [154]. They suggested that MALAT-1's actions in OvCa may include interactions with miRNAs. MiR-503-5p was identified in their investigation as a target gene of MALAT-1 in OvCa. MiR-503-5p expression was significantly downregulated in OvCa cells, according to Sun et al. [154], who hypothesized that the gene might function as a tumor suppressor in this disease. These results imply that MALAT-1 directly binds to miR-503-5p to mediate its biological effects. The research by Sun et al. shed fresh light on MALAT-1's role in OvCa and showed how it may be used as a therapeutic target [154]. There is mounting evidence that miRNAs play an important role in cancer etiology, notably in the modulation of the Janus kinase/signal transducer and activator of transcription 3 (JAK/STAT3) signaling pathway in malignancies [155,156]. The link between MALAT-1 and miR-503-5p in triggering the JAK2/STAT3 signaling pathway was highlighted in the work by Sun and colleagues [154]. These results imply that MALAT-1 may negatively regulate miR-503-5p and activate the JAK2/STAT3 signaling pathway in OvCa cells to increase cell growth and decrease apoptosis.

Moreover, new research from Jin and colleagues reveals that MALAT-1 might affect the PI3K/AKT pathway-mediated EMT in OvCa [29]. According to Jin and colleagues., MALAT-1 controls the Wnt/β-catenin signaling pathway to control the proliferation, migration, and death of OvCa cells. A growth factor called Wnt activates the scaffolding protein and causes the production of Frizzled receptors. Glycogen synthase kinase 3β is phosphorylated, and intracellular signal transduction is started by becoming disheveled. Cell adhesion is increased when the β-catenin protein binds to E-cadherin [157]. Guo and colleagues further showed that MALAT-1 may use the Wnt/β-catenin signaling pathway to control the migration, proliferation, and death of OvCa cells [157]. Furthermore, Zhou and colleagues showed that MALAT-1's effectiveness in accelerating the development of OvCa was mediated by augmenting MMP13 expression and decreasing MMP19 and ADAMTS1 expression [152]. MALAT-1 may be transmitted from OvCa cells to recipient HUVECs through exosomes, according to recent research by Qiu et al. Hu et al. found that metastatic OvCa cells transmit exosomal MALAT-1 to recipient HUVECs, which aids in the formation of angiogenesis. Through triggering several angiogenesis-related genes, such as IL-8, placental growth factor, angiogenin, VEGF-D, VEGF-A, epithelial neutrophil-activating peptide-78, basic fibroblast growth factor, and leptin, the HUVECs are consequently induced to boost angiogenesis [153].

Hu et al. focused on learning more about the function of the ubiquitin E3 ligase MARCH 7 in OvCa [158]. Their research found that MARCH7 communicates with MALAT-1 and controls the expression of ATG7 by out-competing miR-200a. They discovered that MARCH7, significantly when transforming growth factor-β (TGF-β) signaling is present, increases autophagy, invasion, and metastasis in OvCa [158]. The research also showed that TGF-β-SMAD2/3 directly modifies MALAT-1, which in turn controls the expression of MARCH7 and ATG7 [158]. Furthermore, it has been demonstrated that miR-200c suppresses autophagy, improves radiosensitivity, and prevents OvCa cells from migrating and invading [159]. Hu et al. demonstrated that miR-200a prevents OvCa from migrating, invading, and engaging in autophagy. The production of a miR-200a-resistant version of MARCH7, however, partly reversed the observed abnormalities, indicating the function of MARCH7 in controlling cellular autophagy, movement, and invasion [158]. Furthermore, Hu et al. showed that TGF-β causes overexpression of MALAT-1 and that TGF-β suppresses autophagy, migration, and invasion when MALAT-1 is knocked down [158]. They discovered miR-101 and miR-200a as MALAT-1 expression antagonists [158]. Furthermore, it has been found that there is a reciprocal regulation between MARCH 7 and MALAT-1, suggesting that miR-101 plays a role in the modulation of MALAT-1 by MARCH 7 [158]. According to the research, MARCH 7 activates the TGF-β-SMAD2/3 pathway to encourage cellular autophagy, movement, and invasion. The MARCH7, MALAT-1, and ATG7 expression levels were considerably reduced by TGFBR2 suppression or knockdown, demonstrating that MALAT-1 modulates MARCH 7 and ATG7 through the TGF-β-SMAD2/3 pathway [158].

To learn more about the function of MALAT-1 in OvCa, Mao et al. performed in vitro studies using clinical cancer tissues and cancer cell lines [95]. Their research showed that increased expression of MALAT-1 increased drug resistance, invasion, and motility in epithelial OvCa (EOC) cells. This result was made possible by adaptive remodeling techniques that improved tumor cell viability and proliferation while reducing immune responses and apoptosis in the TME, which accelerated EOC tumor growth [95]. MALAT-1 was discovered to be significantly expressed in cancer tissues from various organs, notably EOCs [[160], [161], [162]], which is consistent with earlier studies. Mao and colleagues found that when MYST4 expression was elevated, MALAT-1 lncRNA may have MYST4 histone methyltransferase as a possible downstream target. The lack of a consistent association between MYST4 and MALAT-1 expressions in clinical EOC specimens suggests there may be intricate regulatory systems with unknown mechanisms that need further research [95]. Upregulation of MALAT-1 in EOC cells was associated with increased levels of p-PI3K, *p*-AKT, cyclin D1, vimentin, YAP, and Zinc Finger E-Box Binding Homeobox 2 (ZEB2), as well as a reduction in the expression of E-cadherin. These modifications promoted the growth and spread of tumor cells [95]. Moreover, Mao et al. showed that concurrent excessive expression of MALAT-1 in cancer-associated fibroblasts (CAFs) and EOC cells substantially boosted EOC cell invasion compared to overexpression in either EOC cells or CAFs alone [95]. In conclusion, the findings demonstrate how MALAT-1 overexpression in EOC has an oncogenic function. The discovery of MALAT-1's significance in OvCa brings up new possibilities for investigating personalized medicine and targeted therapeutics in treating this debilitating disease.

### 9.3 Cervical cancer (CC)

Human papillomavirus (HPV) infection is the leading risk factor for the development of CC, which is the fourth most prevalent malignancy in women globally [163,164]. While improvements in HPV vaccination and screening have considerably decreased its prevalence, developing targeted therapeutics will depend on a deeper comprehension of the molecular processes underlying cervical carcinogenesis [165,166]. LncRNAs, particularly MALAT-1, have been identified in this context as possibly major actors in the etiology of CC [167,168]. The molecular processes and clinical importance of MALAT-1 in CC were recently studied by Yang et al. [168]. According to the research, CC tissues had considerably higher levels of MALAT-1 expression than neighboring normal tissues unaffected by the malignancy. This discovery raises the possibility that MALAT-1 contributes to the formation of CC [168]. Additionally, MALAT-1 was shown to be an independent indicator of long-term survival in people with CC. This suggests that MALAT-1 may be a useful biomarker for determining a patient's prognosis for CC [168].

Hao et al. concentrated on the functions of HPV18 E6/E7 and the IL-6/STAT3 signaling pathway to understand the mechanism behind the increased production of MALAT-1 in CC cells [167]. The researchers investigated the interaction of HPV18 E6/E7, IL-6/STAT3, and MALAT-1 in CC HeLa cells [167]. Several cancers, including CC, have been shown to activate the IL-6/STAT3 signaling pathway to support cancer cell survival and invasive expansion [169]. In CC HeLa cells, the findings of this research showed that HPV E6/E7 and the IL-6/STAT3 signaling pathway cooperate together to promote the transcription of MALAT-1. This suggests that viral oncoproteins and cellular inflammatory pathways cooperate in the formation of CC [167]. In keeping with earlier findings and further establishing the modulatory impact of HPV E6/E7 on IL-6/STAT3 signaling, Hao and colleagues found that HPV18 E6/E7 overexpression enhanced IL-6 production and phosphorylated STAT3 in HeLa cells. Additionally, they discovered that HPV18 E6/E7 expression in HeLa cells was reciprocally controlled by IL-6/STAT3 signaling [167]. In HPV16-positive CC cells and in IL-10-induced HPV16 E6/E7 expression, STAT3 has previously been demonstrated to be necessary for HPV16 E6/E7 expression [169,170]. As a consequence, our findings and those of previous research imply a positive feedback relationship between STAT3 signaling and HPV E6/E7. The overexpression of the MALAT-1 gene in CC cells results from a synergistic interaction between HPV18 E6/E7, IL-6/STAT3, and this positive feedback loop [167]. In CC cells, Hao and colleagues showed that IL-6/STAT3 signaling up-regulates MALAT-1. These results support the potential therapeutic usefulness of STAT3 in the cure of cancer by further demonstrating the relationship between active STAT3 signaling and the elevated MALAT-1 expression in cancer cells.

Tie and colleagues looked into the function of MALAT-1 in HPV16-positive CC. MALAT-1 was discovered to be elevated in CC tissues and cell lines that were HPV16-positive [171]. They investigated MALAT-1's molecular processes and biological actions in more detail in CC cells [171]. The findings of this work show that MALAT-1 upregulates MAT2A via sponging miR-485-5p, which in turn enhances the proliferation of HPV16-positive CC cells [171]. Liu et al. discovered that Hsa_circ_0000337 upregulates MAT2A via competitively interacting with miRNA-942-5p and promoting growth, immigration, and invasion in glioma [172]. The liver MAT2A gene was described as an oncogene [173]. The findings indicate that MALAT-1 operates as a ceRNA by sequestering miR-485-5p, thereby facilitating the upregulation of MAT2A. MALAT-1 increases MAT2A expression by connecting to miR-485-5p, which frees MAT2A from miRNA-mediated repression [171]. The research emphasizes MALAT-1 as a possible treatment target for CC that is HPV16-positive. By regulating MAT2A expression by miR-485-5p through MALAT-1, it could be feasible to prevent the growth of CC cells [171].

Liang and colleagues looked into the function of MALAT-1 in the growth of CC and how it interacts with miR-124 [174]. The research discovered an association between CC growth and MALAT-1. This shows that MALAT-1 may be essential for developing tumor cells in CC [174]. MALAT-1 promoted cell colony formation in CC, controlled the cell cycle, and reduced cell apoptosis via sponging miR-145 [175]. These results may prove the role that lncRNA and miRNA play in the development of CC. The expression levels of miR-124 and MALAT-1 were shown to be negatively correlated in cervical carcinoma cells, cervical patient tissues, and mice. This suggests that MALAT-1 and miR-124 might communicate and control each other's expression [174]. Gain- or loss-of-function tests in CC cells used in functional analysis proved that MALAT-1 controls miR-124. It was discovered that MALAT-1 functions as an inhibitory sponge, reducing the expression of miR-124 in cervical carcinoma cells [174]. While transfection with LV-MALAT-1 (MALAT-1 overexpression) abolished the inhibitory impact of miR-124, miR-124 overexpression decreased the growth of CC. This implies that MALAT-1 stimulates the growth of CC by reversing the tumor-suppressive actions of miR-124 [174]. In sum, MALAT-1 has a variety of molecular roles in CC, including controlling cell proliferation, inducing EMT, and activating microRNA sponges. In order to develop successful treatment options targeting MALAT-1 to treat CC, it is essential to comprehend these molecular interactions. Prospective studies in this area may provide viable approaches for enhancing patient outcomes and lessening the burden of CC worldwide.

### 9.4 Breast cancer (BC)

BC is a multifaceted disease that develops and progresses due to several genetic and molecular changes [176,177]. MALAT-1 is one example of a molecule that has recently attracted much interest [178,179]. In a recent study, Ou and colleagues sought to determine the relationship and clinicopathological importance of two biomarkers, MALAT-1 and the transcription factor BTB and CNC homology 1 (BACH1), in TNBC [180]. They statistically evaluated the relationship between the expression of MALAT-1 and BACH1 and their prognostic functions, clinical relevance, and pathological importance in TNBC [180]. The study's findings revealed a strong association between the expression of MALAT-1 and BACH1 and critical clinicopathological factors, such as tumor-node-metastasis stage, metastatic progression, histopathological stage, and survival rates of TNBC patients. Particularly, both the overall survival and disease-free survival of individuals with high MALAT-1 and BACH1 levels of expression decreased [180]. In summary, this research offers insightful information on MALAT-1 and BACH1 expression patterns in TNBC.

The function of MALAT-1 in the progression and prognosis of breast cancer was studied by Zheng et al. [181]. Most significantly, the research showed a negative connection between MALAT-1 expression and the survival rate of BC patients. According to these results, MALAT-1 may be a potential prognostic indicator for BC [181]. According to these results, MALAT-1 could be a crucial biomarker for determining a breast cancer patient's prognosis [181]. The investigators coupled bioinformatics predictions with experimental confirmation to acquire insights into the mechanism behind MALAT-1's role in BC. They found that MALAT-1 binds to a microRNA called miR-339-5p and functions as a ceRNA. The modulation of BLCAP mRNA expression due to this binding relationship shows that MALAT-1 regulates gene expression via its communication with miR-339-5p [181]. In conclusion, this research emphasizes the overexpression of MALAT-1 in BC and its link to a bad prognosis, particularly in certain patient populations. These data propose MALAT-1 as a possible prognostic marker for breast cancer and further our knowledge of the intricate regulatory network, including lncRNAs, miRNAs, and mRNAs, in this malignancy.

Hajibabaei et al. conducted a study that focused on the role of MALAT-1 in the progression of breast cancer via its interactions with miR-561-3p and topoisomerase alpha 2 (TOP2A) [23]. Additionally, highly expressed TOP2A is thought to be implicated in most human malignancies, where its expression is supposed to be increased or dysregulated [182]. MALAT-1, miR-561-3p, and TOP2A expression levels were examined in BC patient specimens and cell lines. In breast cancer specimens and cell lines, it was discovered that MALAT-1 and TOP2A expressions were dramatically elevated, whereas miR-561-3p expressions decreased [23]. Hajibabaei and colleagues showed MiR-561-3p to mediate MALAT-1's effects on BC cell development. MALAT-1 is competitively linked to miR-561-3p, lowering the repression of its target genes like TOP2A, according to results by Hajibabaei and colleagues. MiR-561-3p expression was significantly increased due to MALAT-1 suppression, and TOP2A, a miR-561 target, was subsequently downregulated. In addition, our research discovered that miR-561 and TOP2A had a binding connection in a dual-luciferase reporter gene test [23]. According to their study, miR-561 might act as a sponge for MALAT-1 in BC. Previous studies have identified miR-561 as a tumor suppressor [23]. The mechanistic analysis showed that MALAT-1 functioned as a ceRNA in BC by controlling the miR-561-3p/TOP2A axis. It was demonstrated that MALAT-1 overexpression in BC served as a tumor enhancer by actively sponging miR-561-3p. However, via the miR-561-3p/TOP2A axis, MALAT-1 knockdown was critical in preventing the spread of BC cells [23]. In conclusion, this study emphasizes the importance of MALAT-1 in the development of BC.

In their most recent investigation, Turco and colleagues concentrated on the function of the proteins MALAT-1 and ID4 in controlling VEGFA isoforms in TNBC [178]. They found that MALAT-1 and ID4 stimulate VEGFA exon 7 back-splicing, producing the circular RNA circ_0076611. Circ_0076611 was discovered to be present in TNBC cells, tissues, exosomes produced from TNBC cells, and patient serum [178]. The research showed that circ_0076611 interacts with many transcripts involved in cell proliferation, such as mRNAs for MYC and VEGFA. Circ_0076611 was conducted to encourage cell migration and proliferation in TNBC cells. Circ_0076611 improves the interaction of its target mRNAs with elements of the translation initiation machinery, which improves their ability to express themselves [178]. They discovered that MALAT-1's ID4 protein also contributes to this function [178]. MALAT-1 has been shown to need the ID4 protein to carry out its regulatory function on VEGFA splicing [183]. In addition, ID4 protein is necessary in BC cells for effective communication between MALAT-1 and splicing factor serine/arginine-rich splicing factor 1 (SRSF1) [183]; the latter is in charge of controlling the splicing of VEGFA pre-mRNA [[183], [184], [185]]. Based on the findings of Turco et al., it has been suggested that SRSF1 might exert regulatory control over the back-splicing mechanism occurring on exon 7 of the VEGFA gene [178]. Circ_0076611 is downregulated when SRSF1 is silenced; however, this may or may not be related to the decrease in the VEGF121 isoform that is seen when SRSF1 is absent [183]. In addition, we demonstrated that the whole RNP complex comprising SRSF1 is capable of binding to the polypyrimidine tract binding protein 1 (PTBP1) protein, a negative regulator of circ_0076611 expression, demonstrating the positive function of SRSF1 in the increase of circ_0076611 expression [178]. It makes perfect sense that the RNP complex inhibits PTBP1 activity. In contrast to our findings regarding the expression of circ_0076611, PTBP1 has previously been shown to promote the expression of circRNA_001160 in glioma endothelial cells [186]. Additionally, Circ_0076611 interacts with 321 mRNAs in MDA-MB-468 cells, which could regulate their expression, according to Turco et al. The direct interaction between circ_0076611 and its targets remains uncertain, as it is unclear whether another RNA-binding protein (RBP) facilitates the communication. They discovered VEGFA mRNA as one of the targets of circ_0076611 [178]. The findings of the research demonstrated that circ_0076611 controls VEGFA translation by improving the cooperation between the translation initiation machinery and the VEGFA transcript [178]. Overall, the research showed that circ_0076611 interacts with a number of transcripts involved in cell proliferation, such as the mRNAs for MYC and VEGFA. Circ_0076611 was established to encourage cell movement and growth in TNBC cells. Circ_0076611 improves the interaction of its target mRNAs with elements of the translation initiation machinery, which improves their ability to express themselves [178].

### 9.5 Lung cancer

Lung cancer is estimated to be responsible for the highest number of cancer-related fatalities globally, with an estimated annual mortality rate of approximately 1.6 million individuals [187]. The 5-year overall survival rate is still around 15 %, and patients who do survive this long have a significant incidence of tumor recurrence [188]. In order to facilitate the development of more effective therapeutic approaches, it is imperative to ascertain the oncogenes associated with the initiation and advancement of lung cancer, as well as to gain a comprehensive understanding of the underlying mechanisms involved in this pathological condition. Through various processes, lncRNAs have been demonstrated to play crucial roles in developing malignant tumors [189,190].

Tiansheng et al. looked into the function of MALAT-1 in non-small cell lung cancer (NSCLC) and its underlying processes [191]. Zhang et al. showed that the lncRNA MALAT-1 generated from serum exosomes increased tumor development and motility while decreasing apoptosis in NSCLC [192]. In A549 cells, the expression of MMP2 and MMP9 was also suppressed by MALAT-1 knockdown, along with cell proliferation and invasion. On the other hand, MALAT-1 upregulation promoted NCI–H292 cell growth, invasion, and the production of MMP2 and MMP9. According to these results, MALAT-1 functions as an oncogene in NSCLC [191]. Also, in contrast to normal tissues, our research found that miR-202 was downregulated in NSCLC tissues [191]. In NSCLC tissues, the expression of MALAT-1 is inversely correlated with the expression of miR-202 [191]. The oncogenic impact of MALAT-1 in NSCLC was prevented by overexpressing miR-202, highlighting the critical function of miR-202 in MALAT-1-induced cell growth and metastases in NSCLC cells [191].

The modulatory mechanisms of MALAT-1 in the development of NSCLC were examined in a different study by Song and colleagues [193]. They discovered that MALAT-1 expression was elevated in NSCLC tissues and that its silencing prevented cell growth, migration, and invasion while triggering apoptosis in NSCLC cells [193]. Additionally, it has been shown that MALAT-1 decreases chemosensitivity in NSCLC cells by inhibiting the miR-197-3p/p120 axis [194]. In NSCLC tissues, Song and colleagues identified the downregulation of miR-374b-5p. Functional tests indicated that the upregulation of miR-374b-5p suppressed the progression of NSCLC, migration, and invasion while boosting apoptosis [193]. Further research demonstrated that miR-374b-5p was directly targeting SRSF7. SRSF7 is well recognized for its ability to bind to RNA and engage in alternative pre-mRNA splicing [195,196]. In colon and lung cancer cells, SRSF7 has been linked to a favorable function [197,198]. In summary, the findings indicate that MALAT-1 significantly facilitates tumor proliferation in NSCLC by regulating the expression of SRSF7.

Wang and colleagues investigated the precise mechanism through which MALAT-1 contributes to the development of NSCLC [22]. The results of this study suggest a link between MALAT-1 overexpression and the development of malignant NSCLC. Loss-of-function experiments provided further evidence supporting the inhibitory effects of MALAT-1 inhibition on proliferation, metastasis, and apoptosis in NSCLC. Furthermore, the xenograft mouse model served as an *in vivo* validation of the role of MALAT-1 in promoting tumor growth [22]. The p53 inhibitor murine double minute 4 (MDM4) is commonly overexpressed in various human cancers, suggesting that it might be a therapeutic target for treating these diseases [199]. Furthermore, it has been shown that MDM4 interacts with miRNAs like miR-1205 to enhance the advancement of NSCLC [200]. MDM4 knockdown inhibited cell growth, migration, invasion, and apoptosis, whereas Wang et al. found a positive connection between MDM4 frequency and MALAT-1 levels in NSCLC tissues [22]. MiR-185 has been discovered to limit the growth of tumors and cause G1 cell cycle arrest in NSCLC cells [201,202]. MiR-185-5p levels were shown to be lower in NSCLC, and Wang et al. demonstrated that its overexpression had inhibitory effects on expansion, migration, and invasion, as well as encouraging effects on apoptosis [22]. Functional tests showed that MALAT-1 reverse-modulated miR-185-5p in NSCLC cells, indicating that MALAT-1 is involved in advancing NSCLC via miR-185-5p [22]. By controlling MDM4 expression via miR-185-5p in NSCLC cells, MALAT-1 supported growth, movement, and invasion and decreased apoptosis.

MALAT-1 plays a key role in the initiation and advancement of lung cancer, according to comprehensive studies on the subject. Additional research is warranted to comprehensively elucidate the precise participation of MALAT-1 in the pathogenesis of lung cancer and to explore its potential utility as both a diagnostic biomarker and a target for therapeutic intervention.

### 9.6 The role of MALAT-1 in cancer immunity

Cancer is a complicated and varied illness that often exhibits immune evasion by avoiding immune system monitoring [203,204]. Recent findings show that MALAT-1 influences several immune cells implicated in cancer immunity in addition to its function in cancer cell biology [44,121]. This section examines MALAT-1's effects on immune cells and how they may affect the body's ability to fight cancer.

TAMs are an essential part of the TME, and based on their polarization state, they may have pro- or anti-tumorigenic effects [205,206]. TAMs are often classified into M1 or M2 subgroups, distinguishing them based on functional variances in tumor growth [207]. At the beginning of oncogenesis, TAMs take on an M1-like pro-inflammatory phenotype, triggering an immune response that prevents tumor development. In a recent research investigation, T. Amer and colleagues sought to epigenetically control two immune-modulating proteins, CD80 and mesothelin (MSLN), which are highly expressed in breast cancer and have pro-tumorigenic impacts [208], in order to modulate the anti-inflammatory properties of TAMs in various breast cancer subtypes (hormonal, HER2+, and TNBC). They concentrated on MALAT-1 and HOTAIR as two particular lncRNAs to comprehend their regulatory functions in regulating the tumorigenic activity of TAMs. The researchers investigated the influence of altering MALAT-1 and HOTAIR on the expression of CD80 and MSLN in BC TAMs [208]. Increased expression of CD80 and MSLN was accompanied by downregulation of MALAT-1 and HOTAIR, indicating that these lncRNAs may have cell-specific functions in TAMs [208]. This work suggests a tailored immunotherapeutic strategy to regulate TAM-mediated immune reactions in breast cancer subtypes by epigenetically adjusting the expression of CD80 and MSLN by modifying MALAT-1 and HOTAIR.

NK cells are essential to the body's immune system, notably in detecting and eradicating cancer cells. NK cells play a crucial role in innate immunity, which is significant in fighting cancer [209,210]. In a recent study, Mekky et al. aimed to elucidate the molecular mechanisms underlying the immunogenic properties of the oncogenic MALAT-1 in the TME of patients with TNBC and in TNBC cell lines [44]. The findings showed that MALAT-1 expression was considerably higher in BC patients than in healthy controls, especially in TNBC cases [44]. Immune checkpoints PD-L1 and B7–H4 were expressed less, while NK cell activating ligands MICA/B were expressed more when MALAT-1 was knocked down in MDA-MB-231 cells [44]. When MALAT-1 siRNA-transfected MDA-MB-231 cells and NK cells were cultured together, the cytotoxic capacity of NK cells and CD8^+^ cells was boosted. Both miR-34a and miR-17-5p were shown to decrease in BC patients, and bioinformatics research revealed them as possible targets of MALAT-1. MICA/B levels in MDA-MB-231 cells were raised by miR-34a introduction, while the ectopic expression of miR-17-5p suppressed PD-L1 and B7–H4 checkpoint expressions. The "MALAT-1/miR-34a" and "MALAT-1/miR-17-5p" axes were verified using co-transfection studies, and their influence on the cytotoxic profile of primary immune cells has been established [44]. In summary, their work demonstrates a unique epigenetic change caused by the activation of TNBC cells by the lncRNA MALAT-1. MALAT-1 hampers immune processes within the tumor microenvironment by affecting the miR-34a/MICA/B and miR-17-5p/PD-L1/B7–H4 axes in TNBC patients and cell lines.

The diverse population of immature myeloid cells, known as myeloid-derived suppressor cells (MDSCs), accumulates in the TME and aids in tumor immune evasion [211]. It has been shown that MALAT-1 improves MDSC growth and immunosuppressive capabilities [212,213]. In a recent investigation, MALAT-1 and MDSCs were examined in patients with lung cancer by Zhou et al. [214]. Additionally, it was shown that lung cancer patient PBMCs had lower MALAT-1 levels [214]. The percentage of MDSCs and the relative expression of MALAT-1 were substantially negatively associated. They showed that the fraction of MDSCs rose considerably when MALAT-1 was knocked down, indicating that MALAT-1 adversely regulates MDSCs [214]. These results present the first proof that the lncRNA MALAT-1 regulates MDSCs negatively and decreases peripheral blood mononuclear cells in lung cancer patients. Recognizing how MALAT-1 and MDSCs interact in lung cancer might provide useful information about possible therapeutic targets for controlling the immunosuppressive TME and enhancing the effectiveness of lung cancer therapies. To completely understand the processes by which MALAT-1 modifies immune responses throughout cancer, further study is required, opening the door to creating innovative and successful immunotherapeutic approaches.

### 9.7 Role of MALAT-1 in cancer treatment resistance

One of the main cancer treatments is chemotherapy, which aims to get rid of malignant cells. Chemoresistance, in which cancer cells develop a resistance to the effects of chemotherapy, regrettably continues to be a major obstacle to the curing of cancer. Creating efficient treatment approaches requires a thorough understanding of the molecular processes behind chemoresistance [[215], [216], [217]]. A growing body of data indicates that MALAT-1 is critical in giving resistance to several anticancer therapies [72,179,218]. In this section, we explore the precise ways in which MALAT-1 plays a role in chemoresistance. It is well known that EZH2 increases the methylation of H3K27, resulting in gene silence essential for the development and spread of cancer [219]. Li et al. conducted a study that suggests a potential interaction between EZH2, a protein involved in chromatin modification, and MALAT-1. Due to the inhibition of E-cadherin expression brought on by this connection, MALAT-1 is implicated in the epigenetic control of gene expression, which may further increase chemoresistance [72]. They showed that oxaliplatin-induced EMT and chemoresistance may be reversed by inhibiting MALAT-1 or EZH2. In order to make CRC cells more susceptible to chemotherapy and maybe even overcome chemoresistance, it is suggested that MALAT-1 and its interaction with EZH2 may represent attractive therapeutic targets [72].

A study conducted by Zhang et al. demonstrates through in-vitro functional investigations that the overexpression of miR-22-3p or the inhibition of MALAT-1 could significantly diminish the survival, growth, and drug resistance of gastric cancer cells while concurrently promoting cell death. These findings imply that miR-22-3p and MALAT-1 are key mediators of the GC response to oxaliplatin therapy [220]. They also demonstrate that the effects of MALAT-1 and miR-22-3p on GC drug resistance may be reversed, which is significant. The effects of MALAT-1 knockdown or miR-22-3p increased expression may be offset by blocking miR-22-3p or amplification of ZFP91. This demonstrates the regulatory network's dynamic character and offers possible channels for therapeutic treatments [220]. Through *in vivo* tumor xenograft tests, Zhang et al. further illustrate the function of miR-22-3p in developing GC treatment resistance. This reinforces the conclusions and demonstrates the identified regulatory network's applicability in a scenario with more therapeutic significance [220]. These results provide new opportunities for GC cell sensitization to oxaliplatin therapy and possible therapeutic targets to enhance patient outcomes.

MALAT-1 and SHOC2 are highly expressed in paclitaxel-resistant BC cells, according to research by Shi et al. This raises the possibility that these molecules and treatment resistance in BC may be related [179]. According to the findings, MALAT-1 acts as a sponge for miR-497-5p. A method through which lncRNAs may enclose and disable the activity of certain miRNAs is known as miRNA sponging. In this instance, miR-497-5p works as a sponge for MALAT-1, which stops it from controlling its target genes [179]. Increased expression of MALAT-1 and SHOC2 in BC cells is linked to paclitaxel resistance. Accordingly, the MALAT-1/miR-497-5p/SHOC2 axis may play a role in the emergence of chemoresistance [179]. Patients' responses to paclitaxel therapy are observed to be worse in those with high levels of MALAT-1 and SHOC2. This implies that the concentrations of these compounds may be used as possible biomarkers to anticipate how chemotherapy would affect BC patients [179]. In conclusion, upregulating miR-497-5p may represent a possible therapeutic approach to enhance the paclitaxel treatment response in breast cancer patients. MiR-497-5p has the potential to make breast cancer cells more susceptible to the effects of paclitaxel by blocking both MALAT-1 and SHOC2.

In a recent study conducted by Feng et al., it was discovered that the microRNA miR-200a, which regulates the expression of genes, functions as a sponge for MALAT-1. MALAT-1 sequesters miR-200a, thereby inhibiting its ability to suppress the expression of its target genes through its sponge-like activity. This interaction unveils a novel regulatory axis in the context of lung cancer [218]. The research shows that MALAT-1 promotes ZEB1 expression in lung cancer cells. A transcription factor called ZEB1 participates in the EMT, which is linked to enhanced cancer aggressiveness and treatment resistance [218]. The results imply that focusing on the MALAT-1/miR-200a axis may be a potential therapeutic approach for the management of lung cancer. It could decrease lung cancer cell growth and make resistant cells more susceptible to gefitinib by reducing MALAT-1 or increasing miR-200a activity [218]. This study's main finding emphasizes MALAT-1's molecular relevance to lung cancer cell proliferation and gefitinib resistance. MALAT-1 contributes to medication resistance and cancer aggressiveness by functioning as a miR-200a sponge and encouraging ZEB1 expression.

Radiation therapy, sometimes referred to as cancer radiotherapy, is a popular and efficient treatment option for several forms of cancer. It uses high-energy radiation beams to target and kill cancer cells, causing the least harm to adjacent healthy tissues. Depending on the unique features and stage of the disease, radiotherapy may be used alone or in conjunction with other treatments, such as surgery and chemotherapy. Despite this, it is still challenging to defeat the systems of resistance. Therefore, a more profound comprehension of these molecular pathways will enable scientists to successfully create fresh therapeutic approaches to eliminate cancer [221]. Shen et al. discovered that silencing MALAT-1 significantly impacts the behavior of CRC cells. Enhanced apoptosis and G2/M phase arrest, as well as lower colony survival, proliferation, and migration, are signs that MALAT-1 knockdown in HCT116 cells increases the cells' radiosensitivity. These findings imply that MALAT-1 is essential for developing radioresistance in CRC cells [70]. They propose that MALAT-1's interactions with this particular pathway may allow it to affect radioresistance in CRC [70]. The discoveries advance our knowledge of the molecular causes of CRC radioresistance and open the door to new therapeutic uses that might enhance the prognosis for radiotherapy-treated CRC patients. It is necessary to do further research to clarify the complex molecular relationships at play and to create focused therapies that may make cancer cells more susceptible to chemotherapy and improve treatment results.

## 10 Conclusion

Ultimately, the in-depth investigation of MALAT-1 has shown its important pathogenic molecular function in cancer, including many carcinogenesis phases and the emergence of chemoresistance. According to the research in this review, MALAT-1 is a crucial regulator of cancer development and treatment effectiveness. As MALAT-1 has a variety of impacts on cancer cell proliferation, migration, invasion, and metastasis, it plays a multidimensional role in carcinogenesis. MALAT-1 promotes EMT, angiogenesis, and immune evasion via interactions with important signaling pathways and transcription factors. The acquisition of characteristics resembling cancer stem cells has also been linked to MALAT-1 deregulation, which adds to tumor heterogeneity and therapeutic resistance. The relationship between MALAT-1 and chemoresistance is one of the most prominent features of its functional importance. Increased MALAT-1 levels have been shown to impart resistance to a variety of anticancer treatments, including chemotherapy, targeted therapy, and immunotherapy, according to several studies. To do this, MALAT-1 affects drug efflux pathways, hinders apoptosis, encourages DNA repair, and modifies the TME. To overcome therapy resistance and enhance clinical outcomes for cancer patients, targeting MALAT-1 may offer a potential strategy. Nevertheless, many problems and open issues remain despite the amount of information amassed. First, further research is needed to determine the specific pathways through which MALAT-1 performs its oncogenic effects and imparts chemoresistance. Gaining a comprehensive understanding of the intricate molecular associations involving MALAT-1 may facilitate researchers in enhancing their comprehension of the therapeutic capabilities inherent in this target that drugs can manipulate. Further research is required to examine the diagnostic and prognostic use of MALAT-1 in various cancer types and stages. Moreover, some challenges need to be resolved in producing MALAT-1-targeted medicines. Developing precise and effective delivery systems for MALAT-1 inhibitors or gene editing tools is imperative to enhance treatment efficacy and mitigate off-target effects.

Additionally, developing effective combination approaches offers promise for overcoming treatment resistance and enhancing patient outcomes, such as combining MALAT-1 inhibition with already-approved treatments or immunotherapies. In summary, our knowledge of the intricate regulatory networks involved in carcinogenesis and treatment response has increased due to our developing comprehension of the pathogenic molecular function of MALAT-1 in cancer. MALAT-1 appears as a possible diagnostic biomarker, therapeutic target, and prognostic indication in several cancer types. More research initiatives, including preclinical and clinical trials, are required to transform these discoveries into therapeutically applicable applications and eventually improve cancer patients' care and outcomes. The elucidation of the intricate mechanisms through which MALAT-1 is implicated in the etiology of cancer and its resistance to chemotherapy will facilitate the development of novel therapeutic interventions and personalized treatment strategies in the fight against cancer.

## Ethics approval and consent to participate

Not applicable.

## Consent for publication

All authors have read and agreed to the published version of the manuscript.

## Funding

None.

## Declaration of competing interest

The authors declare they have no conflict of interest.

## CRediT authorship contribution statement

**Dexin Xu:** Writing – original draft, Methodology, Investigation, Conceptualization. **Wenhai Wang:** Writing – original draft, Methodology, Investigation. **Duo Wang:** Writing – original draft, Methodology, Investigation. **Jian Ding:** Validation, Methodology, Investigation. **Yunan Zhou:** Project administration, Methodology, Investigation. **Wenbin Zhang:** Writing – review & editing, Writing – original draft, Project administration, Methodology, Investigation, Conceptualization.

## Abbreviations

HCC: hepatocellular carcinoma
IAPs: inhibitor of apoptosis
BRG1: Brahma-related gene 1
JMJD2C: Jumonji domain-containing protein 2C
CRC-SCs: colorectal cancer stem-like cells
NEAT1: Nuclear Enriched Abundant Transcript 1
Smad1: SMAD family member 1
ZFP91: zinc finger protein 91
OXA: oxaliplatin
PARPi: Poly (ADP-ribose) polymerase inhibitors
PRC2: polycomb repressor complex-2
REST: RE1 Silencing Transcription Factor
CHKA: choline kinase alpha
AR: androgen receptor
CRPC: castration-resistant prostate cancer
TNBC: triple-negative breast cancer
BACH1: BTB and CNC homology 1
MAT2A: Methionine adenosyltransferase 2A
ceRNA: competitive endogenous RNA
GBM: Glioblastoma
TMZ: Temozolomide
MDR: Multidrug resistance
EMT: epithelial-mesenchymal transition
LRP1: lung–resistance related protein 1
NSCLC: non-small-cell lung cancer
MDSCs: Myeloid-derived suppressor cells
MDM4: Mouse double minute 4
YAP: yes-associated protein
ZEB2: zinc finger E-box-binding homeobox-2
TGF-β: Transforming growth factor-beta
ATG7: Autophagy Related 7
ROCK1: rho-associated, coiled-coil-containing protein kinase 1
siRNAs: small interfering RNAs
PD-L1: Programmed death-ligand 1
IPO7: Importin 7
PDAC: pancreatic ductal adenocarcinoma
SRSF1: serine and arginine rich splicing factor 1
mTOR: Mammalian target of rapamycin
LAMP-2: Lysosome-associated membrane protein 2
AHR: Aryl Hydrocarbon Receptor
EZH2: Enhancer of Zeste 2
MMP19: Matrix metalloproteinase-19
ADAMTS1: ADAM Metallopeptidase With Thrombospondin Type 1 Motif 1
